# BAsE-Seq: a method for obtaining long viral haplotypes from short sequence reads

**DOI:** 10.1186/s13059-014-0517-9

**Published:** 2014-11-19

**Authors:** Lewis Z Hong, Shuzhen Hong, Han Teng Wong, Pauline PK Aw, Yan Cheng, Andreas Wilm, Paola F de Sessions, Seng Gee Lim, Niranjan Nagarajan, Martin L Hibberd, Stephen R Quake, William F Burkholder

**Affiliations:** Institute of Molecular and Cell Biology, Agency for Science, Technology and Research, Singapore, 138673 Singapore; Genome Institute of Singapore, Agency for Science, Technology and Research, Singapore, 138672 Singapore; Department of Medicine, Yong Loo Lin School of Medicine, National University of Singapore, Singapore, 117597 Singapore; Department of Gastroenterology and Hepatology, National University Health System, Singapore, 119074 Singapore; Department of Pathogen Biology, London School of Hygiene and Tropical Medicine, London, WC1E 7HT UK; Departments of Bioengineering and Applied Physics, Stanford University and Howard Hughes Medical Institute, Stanford, CA 94305 USA; Visting Investigator, Institute of Molecular and Cell Biology, Agency for Science, Technology and Research, Singapore, 138673 Singapore; Present address: Molecular Biomarkers & Diagnostics, Translational Medicine Research Centre Singapore, Merck Sharp & Dohme, Singapore, 138665 Singapore; Present address: Chugai Pharmabody Research Pte Ltd, Singapore, 138623 Singapore

## Abstract

We present a method for obtaining long haplotypes, of over 3 kb in length, using a short-read sequencer, Barcode-directed Assembly for Extra-long Sequences (BAsE-Seq). BAsE-Seq relies on transposing a template-specific barcode onto random segments of the template molecule and assembling the barcoded short reads into complete haplotypes. We applied BAsE-Seq on mixed clones of hepatitis B virus and accurately identified haplotypes occurring at frequencies greater than or equal to 0.4%, with >99.9% specificity. Applying BAsE-Seq to a clinical sample, we obtained over 9,000 viral haplotypes, which provided an unprecedented view of hepatitis B virus population structure during chronic infection. BAsE-Seq is readily applicable for monitoring quasispecies evolution in viral diseases.

**Electronic supplementary material:**

The online version of this article (doi:10.1186/s13059-014-0517-9) contains supplementary material, which is available to authorized users.

## Background

The ability of viruses to escape host immune responses or develop drug resistance represents a significant challenge to human health. Successful viral evolution is driven by high mutation rates that generate genetically diverse populations within an infected host, which are referred to as viral quasispecies [1,2]. Genetic interactions between mutant viruses within a quasispecies have been proposed to affect the overall fitness of the population through a combination of cooperative and antagonistic effects [3-6]. In recent years, next-generation DNA sequencing technologies have been used to perform ultra-deep sequencing of bulk samples to detect signatures of viral quasispecies by measuring allele distributions of single nucleotide variants (SNVs) [7-10] (this approach is hereafter referred to as 'Deep-Seq'). This approach was recently refined through the development of methods based on redundant sequencing of barcode-tagged or circularized template molecules to reduce the error rates associated with next-generation sequencing, enabling the detection of lower frequency SNVs [11-13]. However, any meaningful attempt to study intra-quasispecies interactions will require the ability to determine viral haplotypes (here, 'haplotype' refers to the set of SNVs that occur on a particular copy of the viral genome) so that the correlation and co-occurrence of SNVs within quasispecies can be characterized. Unfortunately, most sequencing platforms are inherently inadequate with respect to resolving haplotype information beyond several hundred base pairs due to limitations on read length [14], and existing assembly algorithms for haplotype reconstruction from quasispecies suffer from poor sensitivity and specificity [15].

There are several possible approaches for determining haplotypes of viruses with long genomes ('long-range haplotypes'; >1 kb) using existing sequencing technology. One possibility is to use a long-read single-molecule sequencing platform such as the PacBio RS II or nanopore-based sequencers. However, the high intrinsic error rate of the PacBio platform necessitates redundant sequencing across the same template to obtain an accurate consensus sequence, thereby substantially decreasing the effective read length of the technology [16,17]. Sequencers based on nanopore technology are still in development; the accuracy and scalability of this type of technology are currently unclear [18,19]. Another possibility for obtaining long-range haplotypes is to extend the effective single-molecule read length obtained from short-read platforms. To that end, several methods have been developed. A method developed by Hiatt *et al.* [20] relied on tagging individual DNA molecules with a unique sequence, followed by paired-end sequencing of nested breakpoints and performing hierarchical local assembly to reconstruct the template sequence. This method produced 'subassemblies' that were only approximately 700 bp in size due to constraints in the size of molecules that can be efficiently processed on the sequencer. Schwartz *et al.* [21] developed a method that involved stretching long DNA molecules on an Illumina flow cell, followed by *in situ* library construction. Sequence reads that originated from the same molecule were combined by relying on spatial information from the flow cell. This method is technically challenging as it involves customized modification of the sequencer. Single virion sequences have been obtained by molecular cloning or by serial dilution of cDNA or DNA molecules to achieve ≤1 copy per reaction, followed by a 'primer walking' method using capillary sequencing to obtain clonal sequences [22-26]. In principle, this approach can produce high quality haplotypes where sequence length will only be limited by technical constraints in performing molecular cloning and long-range PCR, but suffers from relatively low throughput due to high cost for reagents and labor. Several methods have been developed that rely on physical separation of DNA molecules into reaction chambers containing one or few molecules using limiting dilution or microfluidics, and assembling haplotypes from short reads generated by performing next-generation sequencing on individual reaction chambers [27-30]. Using this approach requires independent library preparation from each reaction chamber, thereby limiting the number of haplotypes that can be obtained per experiment.

Here, we report a method that obtains long haplotypes (>3 kilobases) from viral samples using a short-read sequencer: BAsE-Seq or Barcode-directed Assembly for Extra-long Sequences. BAsE-Seq takes advantage of the low cost-per-base and low error rates of short-read sequencing platforms and addresses the limitations of current methods for extending read lengths. BAsE-Seq relies on attaching unique molecular barcodes to long template molecules, followed by transposing the barcode to random overlapping segments of the template; barcode-tagged sequence reads derived from the same template molecules can be combined to obtain highly accurate haplotype sequences. BAsE-Seq was used to perform single virion sequencing of hepatitis B virus (HBV), which has an approximately 3.2 kb genome and exists as a quasispecies within its host [31-33]. We first describe using BAsE-Seq to obtain individual genome sequences at high accuracy from mixed samples of HBV clones and demonstrate the assembly of viral haplotypes at high sensitivity and specificity. Next, we used BAsE-Seq to obtain the first-time measurement of >9,000 viral haplotypes from a clinical sample. Our method showed good agreement in SNV and haplotype identification when compared with Deep-Seq and clonal sequencing, and allowed us to investigate intra-host phylogenetic structure of HBV quasispecies during chronic infection.

## Results and discussion

### Overview of BAsE-Seq

**Figure 1:**
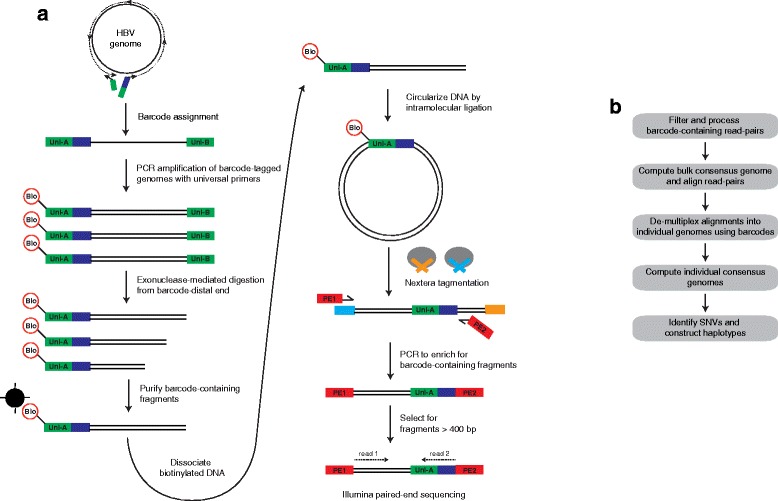
**Outline of BAsE-Seq methodology. (a)** The goal of library preparation is to attach unique barcodes to full-length HBV genomes, and then juxtapose the assigned barcode to random overlapping fragments of the viral genome. A unique barcode is first assigned to each HBV genome using PCR. The two barcode assignment primers contain HBV-specific sequences on their 3′ ends, universal sequences (green) on their 5′ ends, and one of the primers also contains a random barcode (blue). Subsequently, barcode-tagged genomes are clonally amplified by PCR using primers that anneal to Uni-A and Uni-B and that add a biotin label (Bio) to the barcode-proximal end. The barcode-distal end is digested with exonuclease to obtain a broad size distribution of nested deletion fragments. Barcode-containing fragments are purified using Dynabeads, and intramolecular ligation of these fragments yields a library of circular molecules in which different regions of each HBV genome are juxtaposed to its assigned barcode. The circularized molecules are used as a template for random fragmentation and adapter tagging following the Nextera protocol. During PCR enrichment, a set of primers is used to incorporate Illumina-specific paired-end adapters and enrich for barcode-tagged molecules during sequencing. **(b)** Bioinformatics workflow. Barcode-containing read pairs are used to obtain a 'bulk consensus' genome by iterative alignment of read pairs against a GenBank sequence. Aligned read pairs are de-multiplexed into individual genomes based on barcode identity. Consensus base calls are extracted to obtain 'individual consensus' genomes and SNVs are identified in each genome to construct haplotypes.

An outline of the BAsE-Seq methodology is shown in Figure 1a. The basic workflow involves attaching unique barcodes to full-length HBV genomes, and then constructing a library where the barcode is juxtaposed to random overlapping fragments of its assigned genome. Barcode assignment is performed using a pair of primers that contain HBV-specific sequences on their 3′ ends and universal sequences on their 5′ ends (Figure S1 in Additional file 1). Subsequently, barcode-tagged genomes are clonally amplified by PCR using universal primers and exonuclease-mediated digestion is initiated from the barcode-distal end to obtain a broad size distribution of barcode-containing fragments. Next, these fragments are circularized by intramolecular ligation, which juxtaposes different regions of the viral genome adjacent to its assigned barcode. The circularized molecules are used as a template for random fragmentation and adapter tagging using Nextera transposomes, followed by PCR enrichment of the sequencing library to incorporate Illumina-specific paired-end adapters and enrich for barcode-tagged molecules. The library is loaded on a MiSeq for 2 × 150 bp sequencing and a custom sequencing primer is used for the second read to obtain the barcode sequence.

After sequencing, barcode-containing read pairs are used to generate a 'bulk consensus' genome by iterative alignment of read pairs against a HBV reference sequence from GenBank. Next, aligned read pairs are de-multiplexed based on barcode identity, and 'individual consensus' genomes are obtained by extracting the consensus base call at each position. Finally, SNVs relative to the bulk consensus genome are identified from individual genomes and used to construct haplotypes (Figure 1b).

### Developing BAsE-Seq for single virion sequencing

In this subsection, we will discuss the critical steps of our protocol and several challenges that were overcome during the development of BAsE-Seq.

The forward primer used during barcode assignment contains a string of 20 random nucleotides, which allows for approximately 1.1 × 10^12^ possible barcode sequences. Barcode assignment is performed using two cycles of PCR on a template containing 10^6^ double-stranded HBV genomes. This represents an approximately 55,000-fold excess of possible barcode sequences to template molecules; each strand of the genome will be uniquely tagged by a barcode and flanked by universal sequences (Figure S1 in Additional file 1). With this approach, random errors that are introduced subsequent to barcode assignment, such as during library preparation or sequencing, can be removed (Figure S1 in Additional file 1) [13,34]. Hence, the only errors that will remain are PCR errors that were incorporated during barcode assignment or systematic errors that occurred during library preparation or sequencing.

There are two steps in BAsE-Seq that are critical for producing uniform genome coverage: (a) generating deletions from the barcode-distal end of each HBV genome and (b) generating a sequencing library from circularized molecules. In the former, the goal is to obtain an equimolar size distribution of barcode-tagged HBV genomes containing nested deletions from the barcode-distal end, as it will expose different regions along the genome for juxtaposition with the barcode during circularization. This was achieved using a classical approach in which exonuclease III was used for processive digestion from an unprotected end (barcode-distal end) of the template and aliquots were removed at defined time intervals for S1 nuclease digestion to create blunt ends [35]. In the latter, an efficient method was required to fragment circularized molecules and attach sequencing adaptors onto barcode-containing fragments. To achieve this, we relied on a transposase-catalyzed method (Illumina) that is known to introduce slightly higher bias in fragmentation compared with conventional methods, but offered significant advantages in its simplicity and ability to handle low input material [36].

To generate a suitable HBV template for protocol development, we isolated two different HBV clones that contained 17 single nucleotide polymorphisms (SNPs) between them - hereafter referred to as Clone-1 and Clone-2 (Tables S1 and S2 in Additional file 1). During the initial phase of protocol development, we used a 1:1 mixture of Clone-1 and Clone-2 for library preparation in order to assess the ability of our protocol to generate accurate haplotype sequences. Subsequent analysis of sequence data that were generated from the first round of libraries showed that the majority of haplotypes were chimeric, i.e., contained SNPs from both Clone-1 and Clone-2 (data not shown). To identify the steps in our protocol where molecular chimerism was occurring, we prepared libraries in which samples derived from Clone-1 and Clone-2 were mixed at different steps along the protocol. Using this approach, we determined that chimeric sequences were mostly generated during (a) the PCR amplification step that occurred immediately after barcode assignment and (b) the circularization step. Taking reference from previous studies where it was demonstrated that PCR-induced chimeras could be reduced by limiting the number of PCR cycles [37,38], we developed a real-time PCR assay to monitor the PCR efficiency at this step and realized that PCRs that were stopped during the log-linear phase of amplification produced significantly less chimeric sequences. This led us to develop a two-stage PCR protocol to amplify barcode-tagged HBV genomes (further described in Appendix B in Additional file 2) that minimized the formation of PCR-induced chimeras and provided enough PCR products to continue with library preparation. To identify reaction conditions for double-stranded DNA circularization that maximized intramolecular ligation and minimized intermolecular ligation (which will result in the formation of chimeric sequences), we mixed two sub-genomic HBV sequences - each approximately 1 kb long - at equimolar amounts and used them as template for circularization. We developed a quantitative PCR assay (further described in Appendix C in Additional file 2) to measure the abundance of junctions formed by inter- or intra-molecular ligation. This assay allowed us to screen a large number of reaction conditions and identified two key parameters that were critical for optimal circularization: reaction volume and temperature. Notably, a significantly higher rate of intra-molecular ligation (approximately 5%) was achieved by increasing the reaction volume to 45 ml and decreasing the reaction temperature to 10°C. Ultimately, a combination of optimized conditions at both steps - PCR amplification and circularization - allowed us to produce haplotype sequences with minimal chimerism (as presented below).

### Validation of BAsE-Seq with mixed hepatitis B virus clones

To assess the accuracy and sensitivity of BAsE-Seq in performing single virion sequencing on HBV, we mixed Clone-1 and Clone-2 at unequal ratios (1:9 and 1:99) prior to barcode assignment and library preparation, yielding two BAsE-Seq libraries: Lib_1:9 and Lib_1:99. Each library was sequenced on a single run on the MiSeq, producing 6 to 8 million read pairs that could be aligned concordantly to the bulk consensus genome (Table 1; Figure S2 in Additional file 1). Subsequently, each library was analyzed using the 'bulk' approach or the 'individual' genome approach. In the bulk analysis, barcode information was ignored, i.e., sequence reads were not de-multiplexed, and the aligned read pairs were analyzed using a typical pipeline for Deep-Seq in which BAM files were used as input for variant calling using LoFreq [39,40]. In the individual genome analysis, aligned read pairs associated with unique barcodes were analyzed separately as described earlier (Figure 1b).

**Table 1:** **Summary statistics from BAsE-Seq and Deep-Seq of hepatitis B virus**

	**Lib_1:9**		**Lib_1:99**		**S7.1**		
Read-pairs	14,352,128		17,083,497		42,997,995		8,197,770
**Library**	**BAsE-Seq**		**BAsE-Seq**		**BAsE-Seq**		**Deep-Seq**
Type of sample	Mixed clone		Mixed clone		Internal standard	Patient	Patient
Pass-filter read pairs^a^	6,751,411 (47%)		8,816,934 (52%)		545,960 (1%)	26,066,408 (61%)	6,351,796 (77%)
Concordantly aligned^b^	6,027,421 (89%)		8,150,721 (92%)		496,356 (91%)	23,366,358 (90%)	4,261,572 (67%)
High quality genomes	2,390^c^		3,673^c^		345^d^	12,444^d^	
Type of analysis	Bulk	Individual	Bulk	Individual	Individual	Individual	
Median per-base coverage depth	333,677	86	470,036	63	38	45	131,492
True SNVs detected	17 /17	17/17	15/17	17/17		68	
SNVs detected							308
Errors detected	522	218	328	257	11		
Highest per-base error	1.91%	0.202%	2.14%	0.231%	0.69%		
Overall error	0.0524%	0.00674%	0.0324%	0.00541%	0.00214%		

Each BAsE-Seq library was analyzed using the 'bulk' approach, i.e., without de-multiplexing by barcode identity, or the 'individual' approach, i.e., sequence reads associated with unique barcodes were analyzed separately. True SNVs in S7.1 were identified by BAsE-Seq as those that occurred at >0.69% frequency.

^a^Read pairs after removal of adaptor and/or universal sequences. For BAsE-Seq libraries, this only includes read pairs that carry a barcode.

^b^Both reads in a pair were aligned in the expected orientation.

^c^≥ 4 unique reads per base position across ≥85% of the genome.

^d^≥ 4 unique reads per base position across ≥50% of the genome.

At the individual genome level, average per-base coverage was high (>50 unique reads) for both libraries across the majority of bases in the genome (Figure S3 and Table S3 in Additional file 1). We obtained 2,390 and 3,673 high quality genomes - ≥4 unique reads per base position across ≥85% of the genome - from Lib_1:9 and Lib_1:99, respectively (Table 1). These high quality genomes were used in downstream analysis for SNV identification, error analysis, and haplotype analysis.

**Figure 2:**
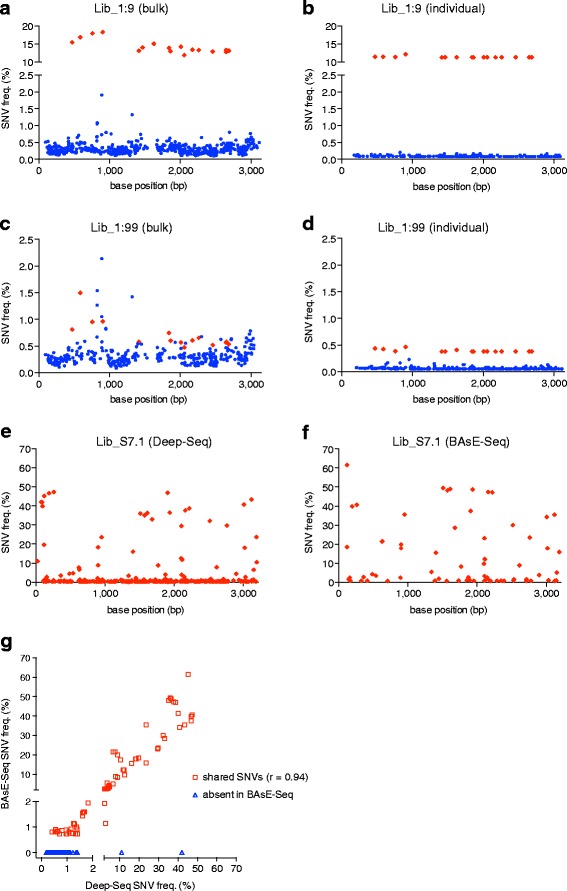
**SNVs in BAsE-Seq and Deep-Seq libraries. (a-d)** SNVs in BAsE-Seq libraries Lib_1:9 and Lib_1:99 were identified as true SNVs (red diamonds) or errors (blue dots) using the 'bulk' approach **(a,c)** or the 'individual' approach **(b,d)**. The frequency of each SNV (y-axis) is plotted against base position in the consensus sequence (x-axis). Additional information is also provided in Tables 1 and 3. **(e,f)** SNVs from S7.1 were identified using Deep-Seq and BAsE-Seq. The BAsE-Seq library contained an internal standard that was used to calculate the error-free frequency cutoff for the library; hence, only error-free SNVs are shown in the BAsE-Seq analysis of S7.1. **(g)** The frequency of SNVs detected in the BAsE-Seq library (y-axis) is plotted against the frequency of SNVs detected in the Deep-Seq library (x-axis). All 68 error-free SNVs identified by BAsE-Seq were also identified by Deep-Seq (Pearson correlation coefficient = 0.94).

The bulk analysis identified all 17 true SNVs at an average minor allele frequency (MAF) of 14.3 ± 1.9% in Lib_1:9 and 15 out of 17 true SNVs at an average MAF of 0.712 ± 0.264% in Lib_1:99. In comparison, the individual genome analysis identified all 17 true SNVs in both libraries, at an average MAF of 11.4 ± 0.2% in Lib_1:9 and 0.394 ± 0.026% in Lib_1:99 (Figure 2). Since the true consensus sequence is known and the error rate of plasmid replication is extremely low (approximately 5 × 10^-10^) [41], variants at non-SNP positions can be classified as errors generated by our protocol; the overall error rate was approximately seven-fold lower in the individual genome analysis (Table 1). Furthermore, the highest per-base error rate is approximately 0.2% in the individual genome analysis, which is approximately nine-fold lower compared with the bulk analysis (Table 1). These results indicate that our consensus base-calling approach using barcodes can substantially reduce the error rate of next-generation sequencing, thereby increasing the sensitivity and specificity of detecting SNVs occurring at ≤2% frequency (Table 1 and Figure 2). Further, using barcodes to tag individual genomes provides more precise estimation of SNV frequencies, as was evident in lower standard deviations in SNV frequencies observed in the individual genome analysis.

Based on the fidelity of the Long PCR Enzyme Mix reported by the manufacturer (7.3 × 10^-6^ errors per nucleotide per PCR cycle), the expected error rate during barcode assignment is approximately 0.0015% - approximately one error in 22 HBV genomes - which sets the expected error rate for BAsE-Seq. However, the error rate for both Lib_1:9 and Lib_1:99 is approximately four-fold higher (Table 1). Interestingly, the errors from individual genomes in both libraries have a significant overlap with regards to base position (*P* < 4e-14, Fisher’s exact test), which suggests that some of the errors were not removed by our approach because they were introduced systematically. It is also likely that the higher than expected error rate could be because the PCR polymerase used during barcode assignment has a higher error rate than is reported by the manufacturer.

For both Lib_1:9 and Lib_1:99, the observed frequencies of the Clone-1 and Clone-2 haplotypes were very close to the expected frequencies (Table 2). In addition to the expected haplotype sequences, two haplotype sequences were detected in each library that differed from the sequences of the two clones used for library preparation. These haplotypes could be the result of molecular chimeras that formed during library preparation. Alternatively, given their low frequency (≤0.05%) and the presence of only one discordant SNV in each haplotype sequence, they could be the result of errors in individual genome sequences. Taken together, these results indicate that BAsE-Seq generates highly sensitive and accurate SNV calls and haplotypes from viral samples.

**Table 2:** **Haplotypes identified by BAsE-Seq in Lib_1:9 and Lib_1:99**

**Haplotypes** ^**a**^	**Lib_1:9**			**Lib_1:99**		
	**Individual** ^**b**^		**Bulk** ^**c**^	**Individual** ^**b**^		**Bulk** ^**c**^
........................................................	2,118	88.6%	85.7%	3,656	99.5%	99.3%
ACACTAAATTTAAACAG	270	11.3%	14.3%	14	0.4%	0.7%
....C................................................	1	0.04%	0%	1	0.03%	0%
ACACTAAATTTAAA...AG	1	0.04%	0%	0	0%	0%
....................A................................	0	0%	0%	2	0.05%	0%
Total	2,390	100%	100%	3,673	100%	100%

^a^Haplotypes observed in individual genomes. The major allele (Clone-2) is represented by a period and the minor allele (Clone-1) is represented by the base at that position (Table S2 in Additional file 2).

^b^Haplotype frequencies observed in individual genomes.

^c^Average allele frequency of SNPs from 'bulk' analysis.

### Evaluation of BAsE-Seq on a patient sample

To evaluate the performance of BAsE-Seq on a clinical sample, BAsE-Seq and Deep-Seq libraries were generated using viral DNA isolated from a chronic hepatitis B patient. This patient sample is hereafter referred to as ‘S7.1’. Sequence reads from the Deep-Seq library were used to generate a bulk consensus genome for S7.1, and subsequent alignment to this bulk consensus genome produced a median per-base coverage depth of 131,492 reads (Table 1; Figure S4 in Additional file 1). We identified 308 SNVs from the Deep-Seq library, ranging in frequency from 0.2% to 47% (Table 1 and Figure 2e). In the BAsE-Seq library, we used an internal standard in order to estimate error frequencies during library preparation and sequencing; the internal standard acts as a control for errors observed in the library that it was prepared from. We prepared the internal standard by assigning barcodes separately to HBV Clone-2; these barcodes contained a two-base insertion that allowed us to distinguish them from patient-specific viral genomes. After barcode assignment, the internal standard was mixed with patient-specific viral genomes and used to build a BAsE-Seq library. Among 345 high quality genomes derived from the internal standard, the highest per-base error rate was 0.69%, which we termed the 'baseline error frequency' for the library. Using the baseline error frequency as a threshold below which a SNV might be due to an error and above which a SNV was treated as a true SNV, we identified 68 true SNVs among the 12,444 high quality genomes assembled from S7.1 (Table 1). The SNVs were evenly distributed across the genome and had a large frequency range: 17 SNVs occurred below 1% frequency, 24 SNVs occurred between 1% and 10% frequency, and 27 SNVs occurred above 10% frequency (Figure 2f). Although our pipeline should preclude SNVs occurring above 50% frequency, one SNV was identified by BAsE-Seq at approximately 62% frequency because the bulk consensus genome was generated using sequence reads from Deep-Seq, where this variant was found to occur at a frequency just below 50%.

There was good agreement between BAsE-Seq and Deep-Seq in SNV identification: all 68 true SNVs identified by BAsE-Seq were also found by Deep-Seq and SNV frequencies were highly correlated between both methods (Figure 2g). Five SNVs at >10% frequency were detected by Deep-Seq but were missed by BAsE-Seq because these SNVs were all located within 60 bp of the BAsE-Seq primer binding sites, where per-base coverage using BAsE-Seq was significantly lower (Figure 2g). All of the remaining SNVs that were only detected by Deep-Seq occurred at frequencies <1.4% (Figure 2g). Among these SNVs, 217 were also found in the BAsE-Seq data but occurred below the baseline error frequency used as a cutoff; it is unclear whether these are true SNVs or errors. The remaining 18 SNVs were not found in the BAsE-Seq data despite good per-base coverage, and are likely to be errors specific to Deep-Seq.

To validate the accuracy of haplotypes observed by BAsE-Seq in S7.1, 20 sub-genomic clones containing the pre-core and basal core promoter region were isolated. Capillary sequencing was performed, which detected a total of five SNVs and five unique haplotypes across a 367 bp region (Table 3). In total, BAsE-Seq analysis of S7.1 identified 2,555 haplotypes with a 100% SNV calling rate across this region. Ten unique haplotypes were detected; four of these haplotypes were also observed in the sequenced clones, which includes the three most frequently observed haplotypes and a haplotype that was detected at approximately 0.08% frequency in BAsE-Seq (Table 3). These results indicate that BAsE-Seq is capable of highly sensitive, accurate and quantitative identification of single virion sequences from a clinical sample.

**Table 3:** **Comparison of haplotypes observed over a 367 bp region in S7.1**

**SNV position** ^**a**^	**118**	**119**	**192**	**258**	**484**		
**Consensus base** ^**b**^	**T**	**G**	**C**	**G**	**C**		
**Variant base**	**G**	**A**	**T**	**C**	**A**		
**Deep-Seq frequency (%)**	**19.6**	**45.2**	**46.7**	**47.3**	**4.5**		
**BAsE-Seq frequency (%)**	**18.5**	**61.5**	**39.9**	**40.6**	**4.2**		
						**Observed in clones**	**Observed in BAsE-Seq**
**Haplotypes** ^**c**^	.	A	.	.	.	10 (50%)	1,588 (62%)
	.	.	T	C	.	2 (10%)	428 (17%)
	G	.	T	C	.	5 (25%)	403 (16%)
	.	.	T	C	A	0	65 (3%)
	G	.	T	C	A	0	27 (1%)
	.	.	.	.	.	0	24 (1%)
	G	A	T	C	.	0	14 (0.5%)
	.	A	.	.	A	1 (5%)	2 (0.08%)
	.	.	T	.	.	0	2 (0.08%)
	G	.	.	C	.	0	2 (0.08%)
	.	A	T	C	.	2 (10%)	0
**Unique haplotypes**						5	10
**Total observed haplotypes**						20	2,555

^a^SNVs identified by all three methods - Sanger sequencing of clones, Deep-Seq and BAsE-Seq.

^b^Base call in the bulk consensus genome.

^c^A period on the haplotype indicates that the position carries the consensus base. SNVs are represented by the identity of the variant base.

### Analysis of haplotypes in the patient sample

Of the 68 SNVs identified by BAsE-Seq in S7.1, 56 confer changes in amino acid sequence, while the other 12 are silent substitutions (Table S4 in Additional file 1). Among the non-synonymous variants, seven are nonsense mutations (one in the open reading frame (ORF) for the HBV C protein, one in the ORF for X protein, and five in the ORF for S protein) and one is a mutation in the stop-codon of the C gene that extends the ORF by six amino acids (Table S4 in Additional file 1); most of these mutations have been previously described [42-44] or exist in sequences from GenBank. Five of the nonsense mutations are located near the end of their ORFs and consequently may reduce or alter the expression or activity of the expressed proteins rather than abolishing expression altogether.

**Figure 3:**
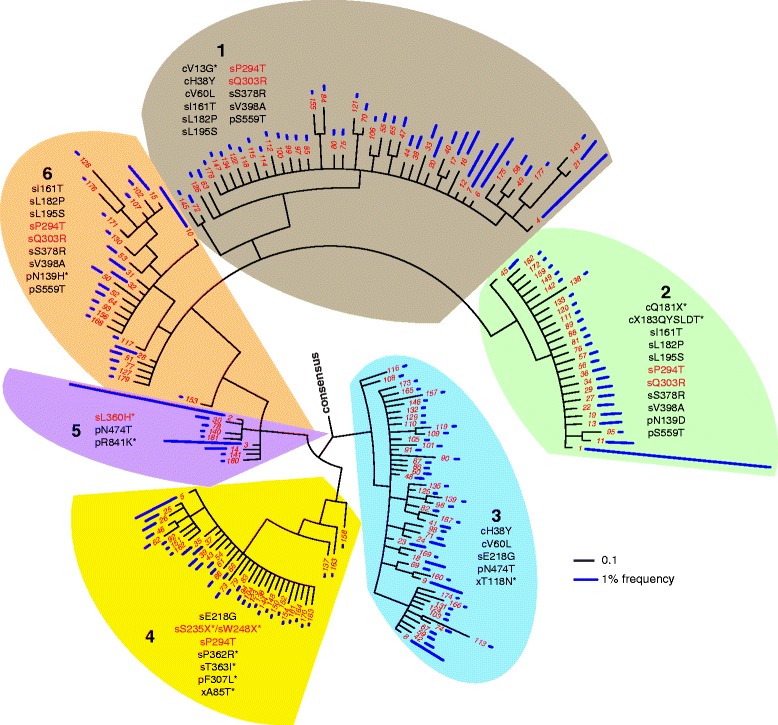
**Phylogenetic analysis of intra-host viral quasispecies.** A phylogenetic analysis of HBV haplotypes identified by BAsE-Seq identified six distinct clades (numbered 1 to 6) in S7.1. The black scale bar represents the expected number of substitutions per site and the blue scale bar represents the frequency at which a particular haplotype was identified in the sample. Amino acid changes that are found in ≥70% of clade members are listed within each clade. Amino acid changes that are unique to each clade are listed with an asterisk. Five out of six clades contain at least one amino acid change (red) that is likely to confer the ability to escape immune detection.

We identified 236 unique haplotypes from among the 9,072 haplotypes assembled from S7.1 that had a 100% call rate across all SNV positions; these haplotypes were detected at frequencies ranging from 0.01% to 8.3%. The actual number of unique haplotypes in the sample is likely to be higher because the library was not sequenced to saturation (Table 3) and any true SNVs present below the baseline error frequency cutoff were not included in the analysis (Table 1). A phylogenetic analysis revealed the existence of at least six distinct clades (Figure 3). Each clade consisted of at least one haplotype sequence that occurred at relatively high frequency and whose sequence is close to the common ancestor of the clade (for example, haplotype 1 in clade 2 and haplotype 5 in clade 4). Some clades have relatively deep branching patterns, which may indicate that they are evolving faster (for example, clade 6). Notably, five out of six clades contained at least one amino acid mutation that is likely to confer a fitness advantage (Figure 3). For instance, haplotypes in clade 4 contain one or more of the following mutations: nonsense mutations (sS235-stop and sW248-stop; refer to Table S4 in Additional file 1 for an explanation of residue numbering) that yield truncated surface proteins that are missing the 'a' determinant, i.e., the immunodominant region of HBsAg [45], and a mutation that results in immune escape [46,47] (sP294T, usually referred to as sP120T, with respect to the small S protein). Also, another immune escape mutation, sQ303R [46,47] (usually referred to as sQ129R), co-exists on the same haplotype with sP294T in clades 1, 2 and 6 and comprises approximately 50% of all haplotypes in the quasispecies. Finally, the sL360H mutation found in clade 5 has been predicted to disrupt homo-dimerization of the S protein [48]. Taken together, the phylogenetic structure of viral quasispecies in this patient is consistent with a scenario where common haplotypes in the founder HBV population gave rise to sub-populations that evolved adaptive mutations required for viral persistence.

Intriguingly, a nonsense mutation (cQ181-stop) and a stop codon mutation (c-stop183QYSLDT) in the C gene are both associated with clade 2 (Figure 3), and co-exist on approximately 93% of haplotypes that carry a mutation at either position in the quasispecies (Table S5 in Additional file 1). Furthermore, a closer inspection of all haplotypes among the S7.1 quasispecies shows that both immune escape mutations described above - sP294T and sQ303R - are found on approximately 98% of haplotypes that contain both cQ181-stop and c-stop183QYSLDT but are only found on approximately 40% of haplotypes that are wild type at cQ181 and c-stop183 (Table S5 in Additional file 1). The co-occurrence of these four mutations on the same haplotypes strongly suggests that cQ181-stop and c-stop183QYSLDT may have arisen on an ancestral genome that already carried the sP294T and sQ303R mutations. A plausible scenario is that cQ181-stop, which removes the last two amino acids from the core protein, may have arisen as an intragenic suppressor of c-stop183QYSLDT to restore the fitness advantage conferred on this haplotype by the immune escape mutations. Importantly, these mutations are separated by >1.3 kb and their occurrence with respect to one another cannot be resolved without long-range haplotype information.

### Future applications and possible improvements to BAsE-Seq

The current manifestation of BAsE-Seq contains a region of approximately 60 bp at each end of the HBV genome where potential SNVs are missed because per-base coverage is significantly lower. This can be resolved by modifying the exonuclease digestion step, such as having additional time points, to allow these regions to be included in library preparation. With some modifications to the protocol that will involve the design of new primer sets, we anticipate that BAsE-Seq can be used to study other DNA viruses (for example, human papillomavirus) or low complexity but genetically heterogeneous regions (for example, B-cell or T-cell receptor sequences). However, further optimization of several steps in the BAsE-Seq protocol - genome amplification, exonuclease digestion and circularization steps - may be required to achieve longer haplotype sequences (>4 kb). By modifying the protocol to attach a barcode during reverse transcription, BAsE-Seq can also be applied to study RNA viruses (for example, HIV-1), or used to estimate the relative abundance of transcript isoforms. Taking into account the technical constraints in performing reverse transcription efficiently across long RNA templates, the future application of BAsE-Seq may be restricted to studying sub-genomic regions of large RNA viruses. Finally, given the input requirements of BAsE-Seq (10^6^ HBV genomes), improvements to the efficiency of barcode assignment and genome amplification will be necessary in order to study clinical samples with low viral load.

### Alternative approaches to BAsE-Seq

Recently, two methods using strategies similar to BAsE-Seq were published. The first method - called Tile-Seq - generated reads up to 3 kb; however, this method did not produce haplotype information because individual template molecules were not tagged separately for downstream analysis [49]. Wu *et al.* [50] developed a method that obtained approximately 1.3 kb viral haplotypes, but relied on performing multiple nested PCRs to generate defined deletions of the initial template; this approach required *a priori* knowledge of the entire template sequence and its specificity in haplotype reconstruction was not demonstrated, thus raising concerns over the presence of chimeric sequences. New computational tools based on haplotype inference of viral quasispecies were recently released and used successfully to construct haplotypes from HIV-1 and hepatitis C virus populations [51,52]; it would be of interest to evaluate their performance on an empirical dataset as we have shown here.

## Conclusions

We have demonstrated that BAsE-Seq successfully performs single virion sequencing on HBV by generating viral haplotypes longer than 3 kb, with substantially improved accuracy in SNV calling compared with conventional deep sequencing. A main advantage of BAsE-Seq over existing computational or molecular-based methods to obtain viral haplotypes is its high sensitivity and specificity. In a mixed HBV clone sample, BAsE-Seq accurately assembled haplotypes present at ≥0.4% frequency and achieved greater than 99.9% specificity. In a clinical sample, a sub-genomic haplotype present at approximately 0.08% frequency was validated by clonal sequencing. Notably, we used BAsE-Seq to obtain the first-time measurement of >9,000 viral haplotypes in a clinical sample, which allowed us to evaluate the intra-host population genetic structure of viral quasispecies in a chronic infection and track the co-occurrence of mutations located several kilobases apart across hundreds of unique haplotypes. The method described here is a significant improvement over existing methods to characterize viral quasispecies and will provide a useful tool to study the population genetic basis of viral persistence in a wide range of infections.

## Materials and methods

### Ethics statement

All patients provided written informed consent according to the Declaration of Helsinki, and the study protocols were approved by the institutional review board of the participating hospitals.

### Hepatitis B virus clones

Viral DNA from a chronic hepatitis B patient was isolated from 100 μl of serum using the QIAamp UltraSens Virus kit (Qiagen, Venlo, Limburg, Netherlands). Full-length HBV amplicons were obtained by PCR amplification of 5 ng of viral DNA using previously published primers [53], gel-purified using a MinElute Gel Extraction kit (Qiagen), cloned into a pCR2.1-TOPO vector (Life Technologies, Carlsbad, CA, USA), and transformed into *Escherichia coli* ABLE K competent cells (Agilent, Santa Clara, CA, USA) following the manufacturers’ protocols. Purified clones were verified for the presence of an approximately 3.2 kb insert by PCR, and full-length sequencing of the insert was performed using a primer walking approach (Table S1 in Additional file 1). The sequencing reactions were performed using a BIGDYE Terminator v3.1 kit (Life Technologies) and loaded on a 3730xl instrument (Life Technologies) for analysis. For BAsE-Seq library preparation, each HBV clone (Clone-1 and Clone-2) was linearized by restriction digest with *NotI* (NEB, Ipswich, MA, USA), gel-purified using a MinElute Gel Extraction kit (Qiagen), quantified using a Qubit dsDNA BR assay kit (Life Technologies), and diluted to 10^6^ copies/μl.

### Patient sample S7.1

S7.1 is a genotype B HBV sample that was isolated from a chronic hepatitis B patient in 1990, and was selected from a database of samples in which clonal sequencing of the precore/core region had been previously described [54]. Briefly, viral DNA was isolated from 200 μl of serum using the QIAamp DNA Blood Mini kit (Qiagen) and nested PCR amplification was carried out on the precore/core region. The 700-bp nested PCR product was purified, cloned into a pGEM-T vector (Promega, Madison, WI, USA), and transformed in *E. coli* JM109 cells (Promega). Positive clones were sequenced using vector-specific primers with BIGDYE Terminator on the 3730xl sequencer (Life Technologies). After quality trimming, a 466-bp region was obtained for each clone for further analysis. Viral DNA from S7.1 was also used for Deep-Seq and BAsE-Seq library preparation. To quantify the number of full-length genomes in the sample, real-time PCR was performed using the EXPRESS SYBR GreenER qPCR Supermix (Life Technologies) with primers (5′-ACTGTTCAAGCCTCCAAGCTG-3′ and 5′-AAAAGTTGCATGGTGCTGGTGA-3′) that amplified full-length amplicons of the HBV genome. The sample was measured in triplicate and its concentration was estimated by plotting the C_t_ values against a standard curve that was generated using a 10-fold dilution series of HBV Clone-2. 10^6^ HBV genomes from the sample were used for BAsE-Seq library preparation.

### BAsE-Seq library preparation

A detailed protocol, including oligonucleotide sequences, is provided as Additional file 2. Using HBV-specific primers that contain universal sequence on their 5′ ends, 10^6^ HBV genomes were uniquely assigned to a molecular barcode (20 random nucleotides) by performing two cycles of PCR using the Long PCR Enzyme Mix (Thermo Scientific, Waltham, MA, USA). Excess primers were removed by Exonuclease I (Enzymatics, Beverly, MA, USA). We clonally amplified 40,000 copies of barcode-tagged genomes using the Long PCR Enzyme Mix (Thermo Scientific) and universal primers, then digested with *SbfI* (NEB) to protect the barcode-proximal end from exonuclease digest. Next, unidirectional nested deletions from the barcode-distal end were generated using a combination of Exonuclease III and S1 Nuclease (Promega) to achieve a broad size distribution of fragments ranging from approximately 300 bp to 3,200 bp. Barcode-containing fragments were purified using streptavidin-coated Dynabeads (Life Technologies) and subjected to end repair using T4 DNA polymerase and T4 Polynucleotide Kinase (NEB). End-repaired molecules were circularized by intramolecular ligation using T4 DNA Ligase (NEB) and uncircularized molecules were removed by digestion with Lambda Exonuclease and Exonuclease I (Enzymatics). After circularization, different regions from each viral genome were juxtaposed with the barcode assigned to that genome. The circularized molecules were used as a template for random fragmentation and adapter tagging using the Nextera XT kit (Illumina, San Diego, CA, USA). During PCR enrichment, a set of custom primers was used to randomly incorporate the ‘P5’ adapter and place the ‘P7’ adapter next to the barcode. Each library was subjected to size selection to remove fragments <400 bp using AMPure XP beads (Beckman Coulter, Brea, CA, USA), verified on a Bioanalyzer (Agilent), and quantified by real-time PCR using a Library Quantification kit (KAPA Biosystems, Wilmington, MA, USA). We loaded 18 pM of each library on a Miseq 300-cycle reagent cartridge (Illumina) for 2 × 150 bp sequencing. During sequencing, a custom sequencing primer that annealed to the forward priming sequence of HBV was used for the second read to ensure that it begins at the barcode.

During library preparation for sample S7.1, barcodes were separately assigned to patient-specific viral genomes and HBV Clone-2 (which we refer to as an 'internal standard'). The barcodes assigned to the internal standard contain a two-base insertion that served to distinguish them from patient-specific barcodes. After barcoding, 5,000 copies of the internal standard were mixed with 35,000 copies of patient-specific genomes and used to build a BAsE-Seq library.

### BAsE-Seq data analysis

Read pairs were trimmed to remove the barcode, universal and adapter sequences using Fastx (v0.0.13) and Trimmomatic (v0.30). After trimming, read pairs that were ≥15 bp in length were aligned to a 'bulk consensus' genome using default parameters on the Burrows-Wheeler Aligner (BWA; v0.6.1). The bulk consensus genome used to analyze the mixed-clone libraries was the Clone-2 reference sequence obtained by Sanger sequencing. For patient sample S7.1, the bulk consensus genome was obtained by first aligning reads using BWA against a genotype B HBV reference sequence (GenBank accession number AF121245.1) and extracting the major base call (or indel) at each position. Then, reads were aligned to the newly derived bulk consensus genome and the process was repeated iteratively until saturation was achieved with the proportion of mapped reads. BAsE-Seq primers amplify the HBV genomic region spanning base positions 42 to 3,156 and 39 to 3,219 on the Clone-2 and S7.1 consensus genomes, respectively.

For 'bulk' data analysis, BAM files from concordantly aligned reads (read 1: forward strand; read 2: reverse strand) were used as input for variant calling using the -Q25 setting (ignore non-reference bases <Q25) in LoFreq [40] (v2.0.0). For 'individual' genome analysis, concordantly aligned reads were de-multiplexed into individual genome BAM files using the identity of the barcode associated with each read pair. PCR duplicates associated with individual genomes were removed using the rmdup function in samtools (v0.1.18) [55]. Next, vcf files were generated using the mpileup (-C50 -Q25 -d 1000 -DEu) function in samtools (v0.1.18). An 'individual consensus' sequence was obtained for each viral genome by extracting the consensus base call at each position. Specifically, a consensus base call was generated if the per-base coverage was ≥4 reads and the concordance was ≥0.7, that is, the major base call occurred in at least 70% of reads. The number of 'high quality genomes' extracted from each sample will depend on the genome coverage threshold (Table S3 in Additional file 1). We used a higher genome coverage threshold (85%) for the mixed clone libraries to maximize the number of haplotypes with complete SNV calls and a lower genome coverage threshold (50%) for the patient sample to maximize the number of haplotypes recovered for downstream analysis. SNVs - relative to the bulk consensus genome - were identified from individual viral genomes and filtered for those that occurred in ≥2 genomes in the sample and where base calls were generated in ≥1,500 genomes at that position. Finally, haplotypes were constructed for each library using the filtered SNVs.

To determine the library-specific error rate for S7.1, read pairs associated with the internal standard were analyzed separately from patient-specific reads. Errors in the internal standard - SNVs relative to the Clone-2 sequence - were identified from individual viral genomes and filtered for those where base calls were generated in ≥100 genomes at that position. The highest per-base error observed in the data was used to set the baseline error frequency for SNVs observed in the patient sample. We defined this as the frequency threshold below which the SNV might be due to an error and above which the SNV is treated as a true SNV.

To maximize the recovery of haplotype information, we implemented a method to impute the identity of ambiguous bases (Ns) in haplotypes that may arise as a result of low per-base coverage or concordance. First, information was shared across haplotypes using a conservative clustering approach. Haplotypes were clustered using a greedy algorithm that sequentially builds a set of seeds (for clusters) considering haplotypes in order of decreasing abundance. Haplotypes that perfectly matched one of the existing seeds (ignoring Ns) were clustered with them and used to generate a consensus haplotype. Consensus bases were determined by taking the most common base if its frequency was at least 10% greater than the next most common base and using an N otherwise. Haplotypes with Ns in more than 10% of their bases and less than two non-reference bases were excluded from consideration as potential seeds and were reported as singleton clusters.

Unless otherwise stated, custom perl and shell scripts were used for data analysis and are available upon request.

### Phylogenetic analysis

Haplotypes with less than 10 Ns and count ≥10 were used to construct a phylogenetic tree using MrBayes [56] (version 3.2.2; consensus based on 10,000 sample trees, GTR model, γ-distributed rate variation, burn-in of 100,000 iterations and sampling every 200 iterations) and plotted in R using the Ape package [57].

### Deep-Seq library preparation

HBV-specific primers (5′-GCTCTTCTTTTTCACCTCTGCCTAATCA-3′ and 5′-GCTCTTCAAAAAGTTGCATGGTGCTGG-3′) were used to generate a full-length amplicon of the HBV genome using the PfuUltra II Fusion HS DNA Polymerase (Agilent) according to the manufacturer’s instructions. The PCR product was run on a 1% agarose gel and the approximately 3.2 kb fragment was purified using the QIAquick Gel Extraction Kit (Qiagen). The purified sample was sheared into 100 to 300 bp fragments using the following conditions on the Covaris S2 (Covaris, Woburn, MA, USA): duty cycle, 20%; intensity, 5; cycles per burst, 200; time, 110 seconds. The fragments were purified using the QIAquick PCR purification kit (Qiagen) and a Deep-Seq library was prepared using the KAPA Library Preparation Kit (KAPA Biosystems) following the manufacturer’s instructions. TruSeq adapters and dual-indexing primers (Illumina) were used for library preparation and the final PCR step was performed using PfuUltra II Fusion HS DNA Polymerase according to the manufacturer’s instructions. The library was quantified by real-time PCR using a Library Quantification kit (KAPA Biosystems) and loaded on a flowcell for 2 × 101 bp sequencing on a HiSeq 2500 (Illumina).

### Deep-Seq data analysis

The essential steps in our data analysis pipeline follow the protocol described in Aw *et al*. [39]. In brief, a consensus sequence for the sample was obtained by iterative alignment of the sequence reads against a reference using BWA (as described above). After the final mapping, SNVs were identified using LoFreq [40] (v2.0.0) and variants within the primer region (base positions 1 to 21 and 3201 to 3220) were removed.

### Data availability

Raw data have been deposited in the NCBI Sequence Read Archive under accession number PRJNA251790.

## Additional files

Additional file 1: Figure S1. Barcode assignment and error removal. **Figure S2.** Allele frequency in mixed-clone libraries **Figure S3.** Coverage depth for individual genomes. **Figure S4.** Per-base coverage in S7.1 using Deep-Seq. **Table S1.** Primers used for sequencing HBV clones. **Table S2.** SNPs between Clone-1 and Clone-2. **Table S3.** Genome coverage per sample by BAsE-Seq. **Table S4.** True SNVs identified by BAsE-Seq in S7.1. **Table S5.** Haplotype analyses of immune escape mutations. Additional file 2:
**BAsE-Seq detailed protocol.**

## Abbreviations

BAsE-Seq: Barcode-directed Assembly for Extra-long Sequences
bp: base pair
BWA: Burrows-Wheeler Aligner
HBV: hepatitis B virus
MAF: minor allele frequency
ORF: open reading frame
PCR: polymerase chain reaction
SNP: single nucleotide polymorphism
SNV: single nucleotide variant
