# Supplementary material for: BAsE-Seq: a method for obtaining long viral haplotypes from short sequence reads
Source: Genome Biol. 2014 Nov 19;15(11):517. doi: 10.1186/s13059-014-0517-9 (PMC4269956; doi:10.1186/s13059-014-0517-9)
Supplement: Additional file 1: Figure S1. — Barcode assignment and error removal. Figure S2. Allele frequency in mixed-clone libraries Figure S3. Coverage depth for individual genomes. Figure S4. Per-base coverage in S7.1 using Deep-Seq. Table S1. Primers used for sequencing HBV clones. Table S2. SNPs between Clone-1 and Clone-2. Table S3. Genome coverage per sample by BAsE-Seq. Table S4. True SNVs identified by BAsE-Seq in S7.1. Table S5. Haplotype analyses of immune escape mutations. [file 13059_2014_517_MOESM1_ESM.pdf]

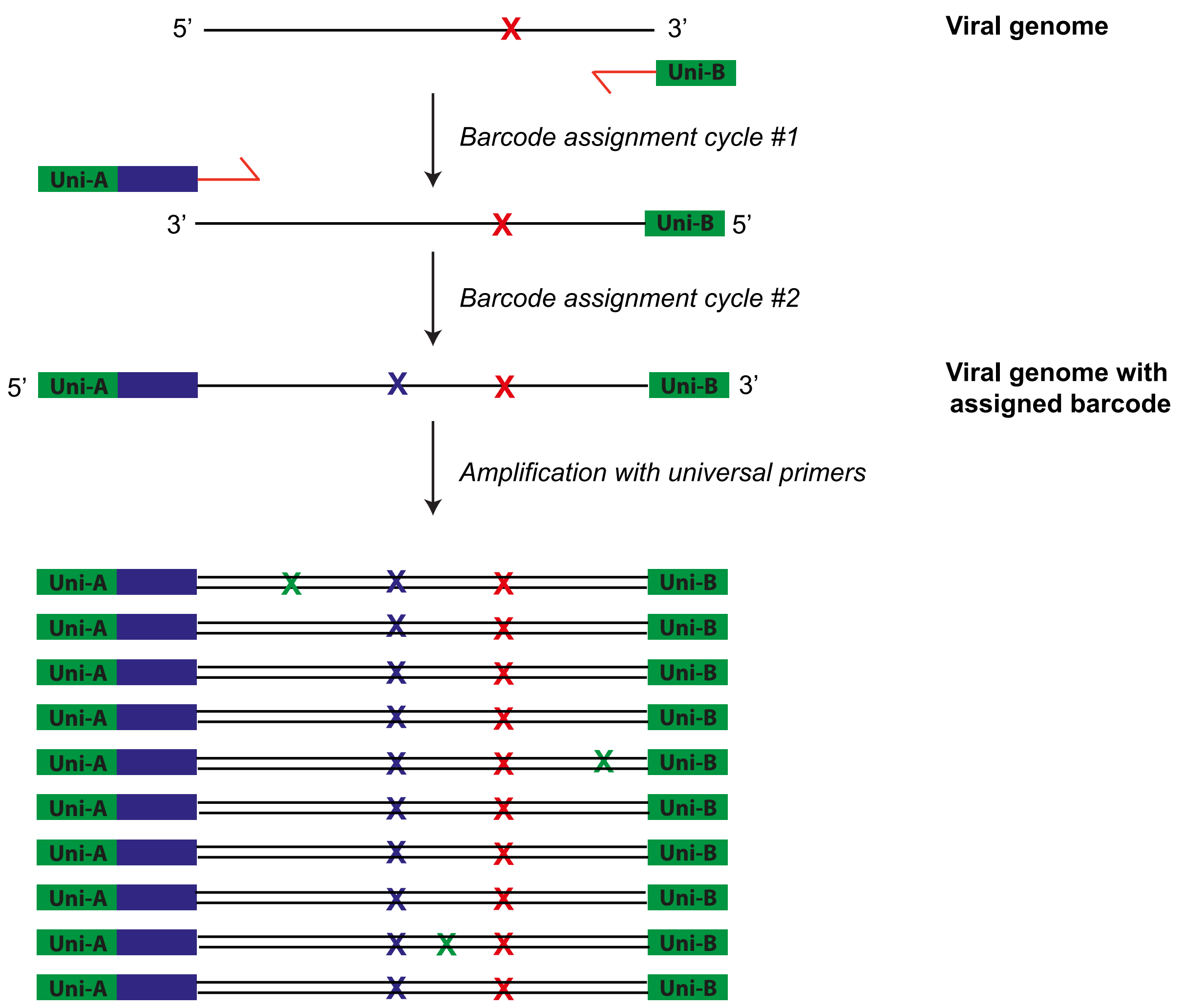

Figure S1. Barcode assignment and error removal.
Each viral genome is assigned to a random barcode, which serves as a unique identifier for sequences originating from each genome. Barcodes also perform another function, allowing for the identification and subsequent removal of the vast majority of errors introduced by next-generation sequencing. Briefly, barcode assignment primers carrying universal sequences (green rectangle; Uni-A or Uni-B) on their 5'-end will anneal to opposite ends of each genome. One of the primers will also carry a barcode (blue rectangle). After two rounds of PCR, each genome will be tagged with a unique barcode. Next, barcode assignment primers are removed by exonuclease digestion, followed by PCR using universal primers to generate whole-genome amplicons. After PCR amplification, mutations that pre-existed on the template (red cross) or errors introduced during barcode assignment cycle #1 or #2 (blue cross) will be found in all the molecules that are associated with a particular barcode. In contrast, errors introduced in subsequent steps of library preparation, sequencing, or base-calling (green crosses) can be easily identified because they will only be present in a minority of these molecules.

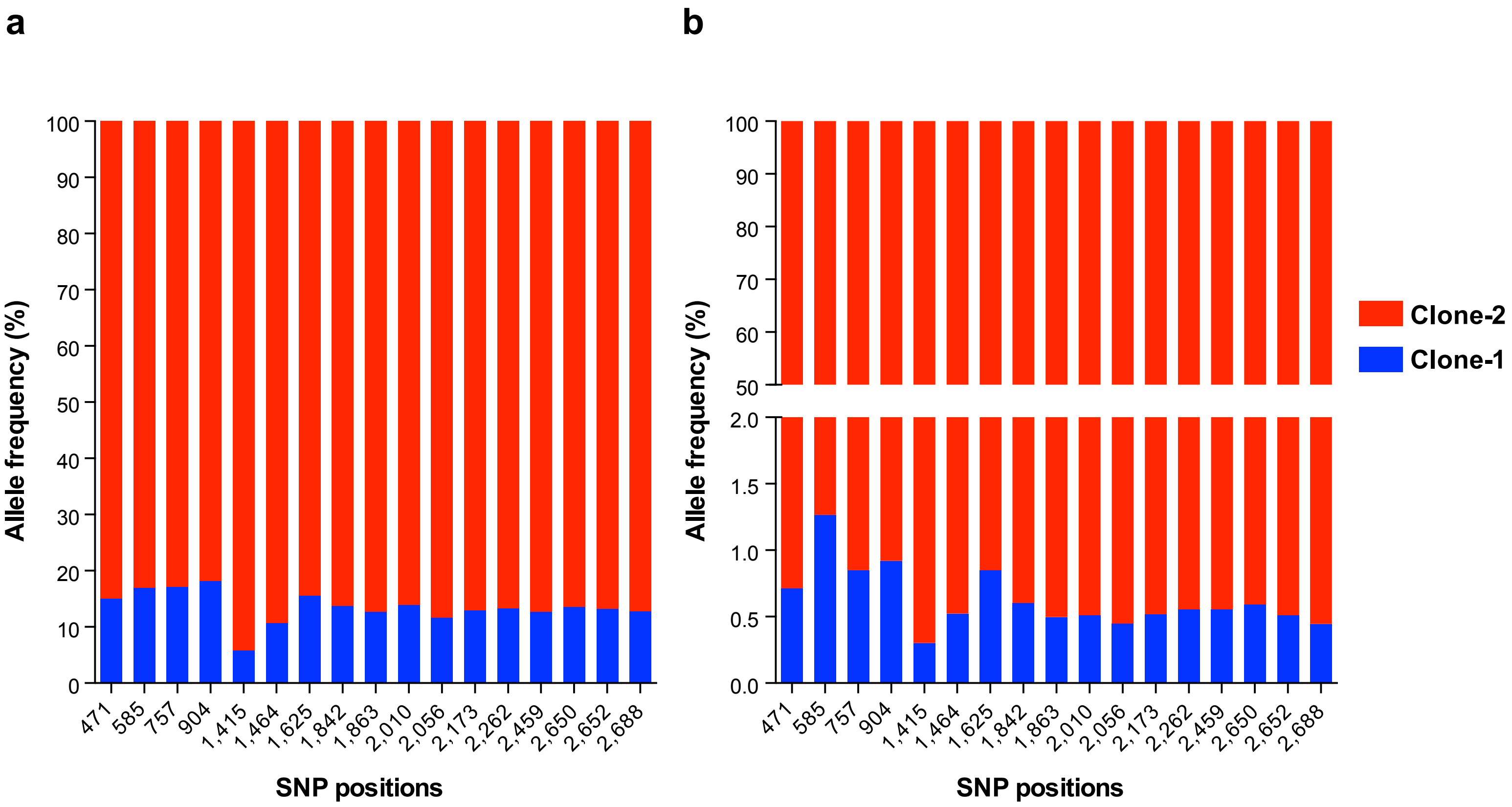

Figure S2. Allele frequency in mixed-clone libraries
HBV Clone-1 and Clone-2 were mixed at (A) 1:9 or (B) 1:99 ratios prior to barcoding and preparation of BAsE-Seq libraries. In each library, ≥Q25 bases from bulk data were used to determine allele frequencies at each of the 17 SNP positions. The sequence representation in each library was very close to the mixing ratio.

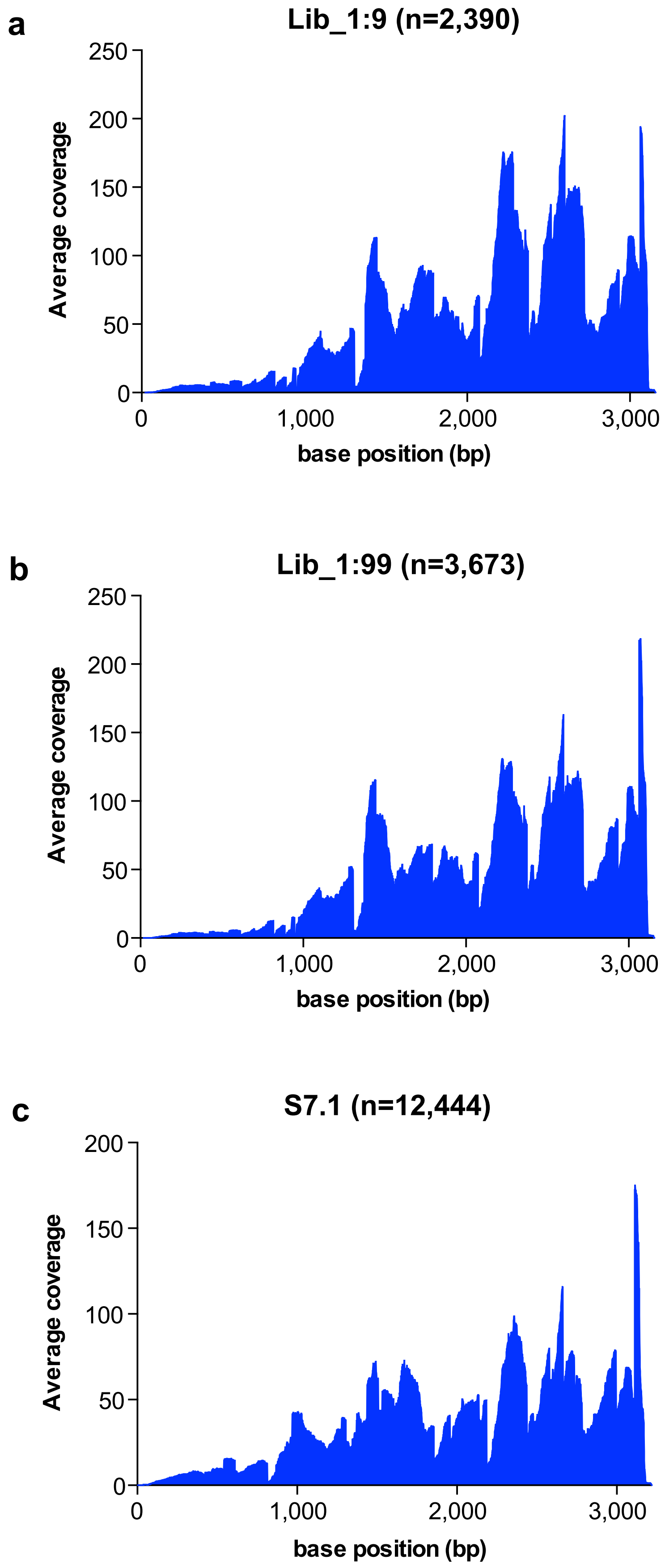

Figure S3. Coverage depth for individual genomes.
Average per-base coverage depth (y-axis) is plotted against base position in the consensus sequence (x-axis) for high coverage genomes in (A) Lib_1:9 (genomes with ≥85% coverage) (B) Lib_1:99 (genomes with ≥85% coverage) and (C) S7.1 (genomes with ≥50% coverage). The number of genomes in each library is shown in parentheses.

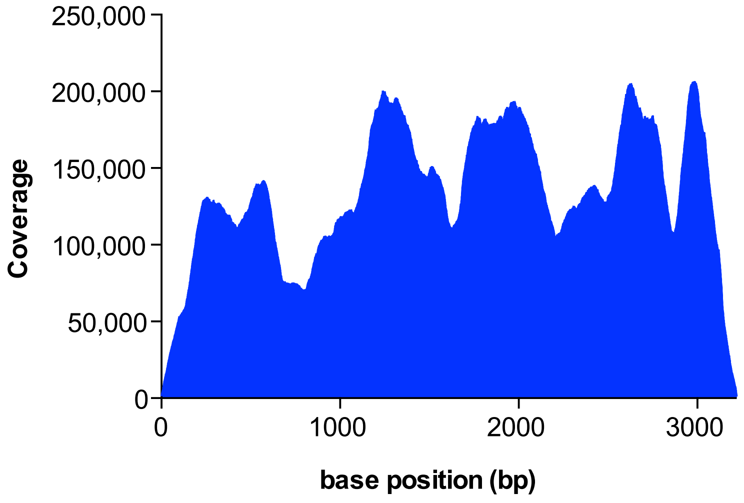

Figure S4. Per-base coverage in S7.1 using Deep-Seq
Sequence reads from a Deep-Seq library from clinical sample S7.1 were aligned to the sample-specific consensus sequence and total read depth per base position was plotted across the genome.

Table S1. Primers used for sequencing HBV clones.

Primer	Sequence
M13 Forward	GTAAAACGACGGCCAG
M13 Reverse	CAGGAAACAGCTATGAC
HBV_P3C6_F2	GGACTCACAAGGTGGGAAAC
HBV_P3C1_F3	ACGAATCTTTCTGTTCCCAATCC
HBV_P3C6_R2	CAGCAAACACTTGGCAGAGA
HBV_P3C1_R3	GGTGAGTGATTGGAGGTTGG

Table S2. SNPs between Clone-1 and Clone-2.

Position in clone[1]	Clone-1	Clone-2
471	A	C
585	C	T
757	A	G
904	C	A
1415	T	C
1464	A	G
1625	A	G
1842	A	G
1863	T	C
2010	T	C
2056	T	G
2173	A	C
2262	A	C
2459	A	C
2650	C	A
2652	A	C
2688	G	A

[1]Position of each SNP is shown with respect to Clone-2 sequence.

Table S3. Genome coverage per sample by BAsE-Seq.

Coverage[1]	Number of genomes		
	Lib_1:9	Lib_1:99	S7.1
≥50%	4,035	6,883	12,444
≥75%	2,844	5,643	8,302
≥85%	2,390	3,673	6,840
≥95%	1,122	382	2,465

[1]Percentage of bases in each viral genome with a coverage depth of ≥4 unique reads.

Table S4. True SNVs identified by BAsE-Seq in S7.1.

Base	Consensus	Variant	SNV freq. (%)	Amino acid change[a]	
118	T	G	18.5	cV13G	
119	G	A	61.5	-	
143	T	C	1.6	-	
156	T	G	2.4	cS26A	
163	G	A	0.8	cR28Q	
192	C	T	39.9	cH38Y	
258	G	C	40.6	cV60L	
326	G	A	0.8	-	
358	T	C	2.8	cM93T	
413	G	A	0.9	-	
484	C	A	4.2	cP135Q	
537	G	T	3.5	cG153C	pE17D
624	C	T	21.4	cQ181-stop	-
630	T	C	21.6	c-stop183QYSLDT	-
705	A	T	0.8		pK73N
892	T	C	2.4		pY136H
901	A	C	17.9		pN139H
901	A	G	19.8		pN139D
951	G	A	35.6		-
1342	T	A	5.5	-	pY286N
1390	G	A	0.9	-	pV302M
1405	T	C	15.6	-	pF307L
1448	T	C	1.9	sF141L	pL321P
1509	T	C	49.4	sI161T	-
1562	G	A	0.7	sA179T	pR359H
1572	T	C	48.2	sL182P	-
1611	T	C	48.9	sL195S	-
1680	A	G	28.6	sE218G	-
1731	C	A	0.9	sS235-stop	-
1770	G	A	8.2	sW248-stop	-
1852	A	C	1.1	sQ275H	-
1875	T	A	2.6	sL283Q	-
1875	T	C	1.4	sL283P	-
1894	A	G	1.1	-	pN470D
1907	A	C	37.5	sP294T	pN474T
1935	A	G	48.8	sQ303R	-
1953	C	A	0.8	sP309H	-
2100	T	C	9.7	sV358A	-

2106	T	A	0.8	sL360H	-
2106	T	C	23.1	sL360P	-
2109	C	T	3.2	sS361F	-
2112	C	G	12.2	sP362R	-
2115	C	T	12.1	sT363I	-
2145	G	A	2.5	sW373-stop	-
2151	G	A	1.9	sW375-stop	-
2161	T	A	47.3	sS378R	pS559T
2196	T	G	0.7	sL390-stop	-
2220	T	C	47.2	sV398A	-
2289	G	A	0.8		-
2346	G	A	0.9		-
2391	A	C	0.7		-
2517	A	G	30.0		-
2526	G	A	0.9		-
2533	C	A	1.6		pH683N
2616	A	G	8.9		pI710M
2712	A	G	1.6		-
2715	A	C	0.7		pK743N
2763	A	C	23.5		-
2778	A	G	0.8	xR4G	-
2899	T	C	3.8	xV44A	-
3008	G	A	34.3	-	pR841K
3021	G	A	17.9	xA85T	
3033	T	C	1.1	-	
3121	C	A	35.5	xT118N	
3123	G	A	0.7	xE119K	
3147	G	A	1.0	xV127I	
3154	T	C	5.1	xL129S	
3190	T	A	16.0	xL141-stop	

[a]Abbreviations of HBV proteins: p = polymerase; c = core; s = surface; x = X. Silent substitutions in a particular open reading frame are denoted as '-'. A stop codon is denoted as '-stop'. Amino acid changes in polymerase are shown in a separate column as the ORF for the polymerase gene overlaps the c, s and x genes. Amino acid coordinates for all four proteins are based on the annotation for accession number AB219428.1. Amino acid coordinates for the surface protein are based on the large S protein (pre-S1 + pre-S2 + S).

Note: The nomenclature for "amino acid changes" follows the following format: protein-consensus-coordinate-variant, where consensus refers to the majority amino acid and variant refers to the minority amino acid in the quasispecies. The only exception is the amino acid change at base position 1907 (sP294T), where the proline (P) residue is a minority variant in the sample. This was done to reflect the occurrence of a well-described immune escape mutation (P to T) at this position. In other words, the majority of the virions in the quasispecies carry the immune escape mutation.

Table S5. Haplotype analyses of immune escape mutations.

Genotype at core gene	Number of haplotypes	Haplotypes with both immune escape mutations (sP294T + sQ303R)	
Wild type[a]	9,168	3,649	40%
Nonsense mutation only (cQ181-stop)	148	83	56%
Stop-codon mutation only (c-stop183QYSLDT)	13	3	23%
Both mutations (cQ181-stop + c-stop183QYSLDT)	2,152	2,101	98%

[a]Wild type at both cQ181 and c-stop183.
