# Supplementary material for: BAsE-Seq: a method for obtaining long viral haplotypes from short sequence reads
Source: Genome Biol. 2014 Nov 19;15(11):517. doi: 10.1186/s13059-014-0517-9 (PMC4269956; doi:10.1186/s13059-014-0517-9)

BAsE-Seq Detailed Protocol

Barcode-directed **As**sembly for **E**xtra-long **Seq**uences

Last updated: 2 July 2014

Overview of methodology

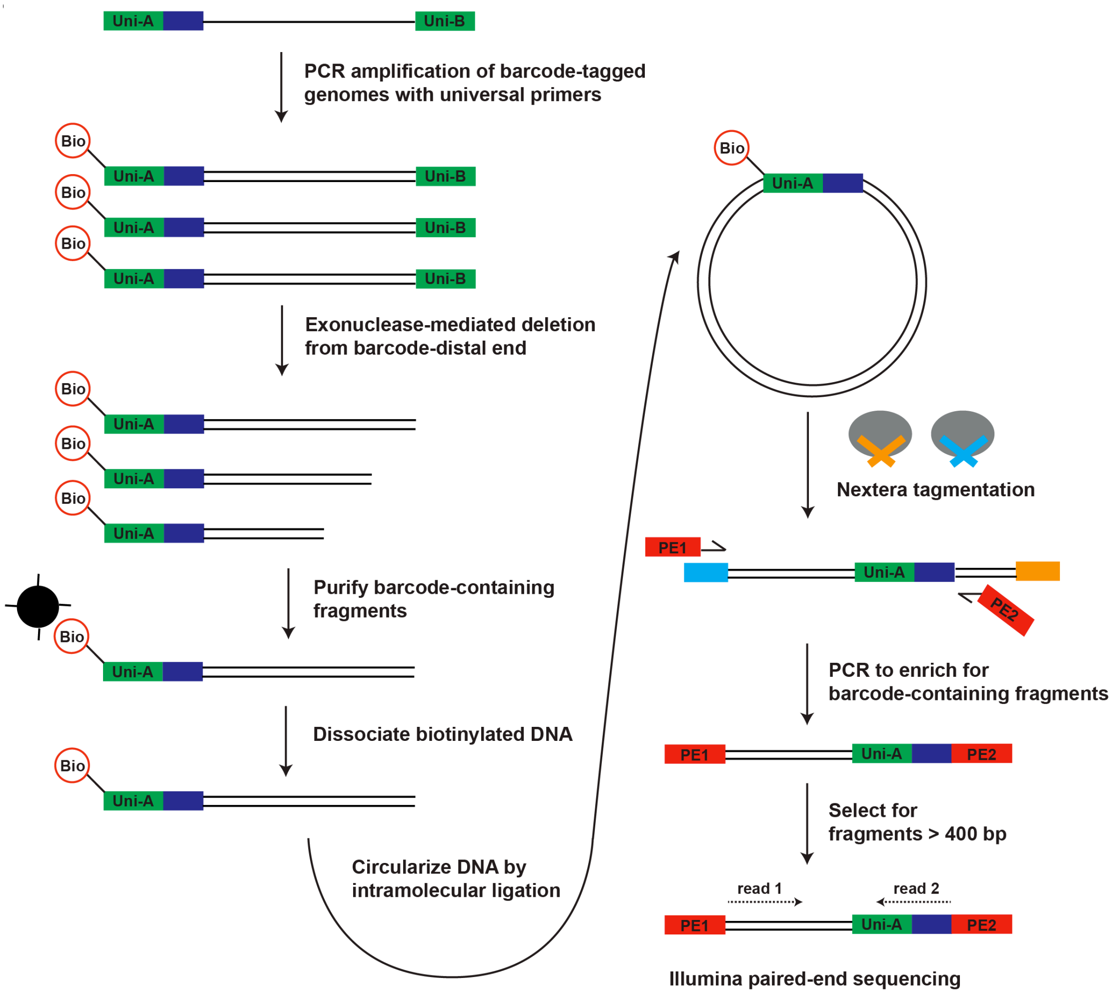

Summary

This protocol for library preparation is currently in use by the Microfluidics Systems Biology Lab at the Institute of Molecular and Cell Biology (IMCB) in the Agency for Science, Technology and Research (A*STAR, Singapore) and is an adaptation of BAsE-Seq to perform single virion sequencing on the Hepatitis B virus (HBV) using the Illumina sequencing platform.

The goal of library preparation is to tag overlapping fragments of each viral genome with its assigned barcode in order to obtain uniform sequence coverage. In the first step, individual viral genomes are uniquely assigned to a DNA barcode. Subsequently, clonally amplified barcode-tagged molecules are deleted from the barcode-distal end to achieve a broad size distribution of fragments ranging from ~300 bp to 3200 bp. Next, barcode-containing fragments are purified and subjected to

end repair. The end-repaired molecules are circularized by intramolecular ligation. After circularization, different regions from the viral genome will be juxtaposed to its assigned barcode. The circularized molecules will be used as template for random fragmentation and adaptor tagging using the Nextera XT kit (Illumina). The primers used during PCR enrichment of the sequencing library will be designed such that the second sequencing read will start at the barcode. Finally, the PCR products are subjected to size selection before sequencing. A custom sequencing primer that anneals to the forward priming sequence of HBV is used for the 2nd sequencing read.

Reagents

EXPRESS SYBR GreenER qPCR Supermix (Life Technologies 11784-01K)
SYBR Green I (Lonza 50513)
ROX Reference Dye (Life Technologies 12223-012)
Nuclease-free water (Promega P1193)
Agencourt AMPure XP (Beckman Coulter A63881)
Long PCR Enzyme Mix (Thermo Scientific K0182)
dNTP mix (NEB N0447L)
Exonuclease I (Enzymatics X801L)
SbfI-HF (NEB R3642L)
Exonuclease III (Promega M1815)
S1 Nuclease (Promega M5761)
T4 DNA Polymerase (NEB M0203L)
T4 Polynucleotide Kinase (NEB M0201L)
T4 DNA Ligase (NEB M0202L)
Lambda Exonuclease (Enzymatics X8030L)
Qubit dsDNA assay kits (Life Technologies Q32850 and Q32851)
Agilent DNA 7500 Kit (Agilent 5067-1506)
Agilent High Senstivity DNA Kit (Agilent 5067-4626)
Dynabeads kilobaseBINDER kit (Life Technologies 60101)
MinElute PCR purification kit (QIAGEN 28004)
GlycoBlue (Life Technologies AM9516)
Nextera XT Sample Preparation Kit (Illumina FC-131-1096)
Nextera XT Index Kit (Illumina FC-131-1001)
MiSeq Reagent Kit v2 300 cycle (Illumina MS102-2002)
Library Quantification Kit – Illumina/Universal (KAPA Biosystems KK2824)

Equipment

1.5 mL "LoBind" microcentrifuge tubes (Axygen MCT-150-L-C)
0.2 mL PCR tube with flat cap (Axygen PCR-02-C)
0.2 mL 8-strip PCR tubes (Axygen PCR-0208-C)
50 mL centrifuge tubes (Labcon 3191-870-008)
QPCR 96-well plate, non-skirted (Agilent 401333)

Standard PCR thermocycler (Bio-Rad DNA Engine Tetrad 2)
Real-time PCR system (Stratagene Mx3005P)
Magnetic stand; DyMag-2 (Life Technologies 12321D)
Eppendorf thermomixer R (VWR 460-1112); 24 x 1.5 ml thermoblock (460-1114)
2100 Bioanalyzer (Agilent)
Microcentrifuge (Eppendorf 5424)
Multipurpose centrifuge with 6x85 ml rotor (Eppendorf 5810R and F-34-6-38)
Sample Mixer/Rotator (Life Technologies 15920D)
Standard water bath
SpeedVac (Eppendorf EPPE5305000.169)
Qubit 2.0 Fluorometer (Life Technologies Q32866)

Oligos

All oligos were ordered from Integrated DNA Technologies (IDT) and were HPLC-purified unless otherwise stated.

**Oligo**	**Sequence**
HBV_Hong_F (desalted)	ACTGTTCAAGCCTCCAAGCTG
HBV_Hong_R (desalted)	AAAAGTTGCATGGTGCTGGTGA
A-SbfI-ID20-HBV_For (Ultramer)	ATGCTGCCTGCAGGATGTCGNNNNNNNNNNNNNNNNNNNNACTGTTCAAGCCTCCAAGCTG
A-SbfI-ID20v2-HBV_For (Ultramer)	ATGCTGCCTGCAGGATGTCGATNNNNNNNNNNNNNNNNNNNNACTGTTCAAGCCTCCAAGCTG
B-HBV_Rev	GTTCGGTAACTGGAGCTGACAAAAGTTGCATGGTGCTGGTGA
Uni-A-SbfI-Bio	ATGCTGCCTGCAGGA*/iBiodT/*G*T*C*G
Uni-B	GTTCGGTAACTGGAGCTGAC
NexteraXT-PE1	AATGATACGGCGACCACCGAGATCTACACTAGATCGCTCGTCGGCAGCGTC
Illumina-PE2_HBV-For.RevComp	CAAGCAGAAGACGGCATACGAGATGAACCGCTCTTCCGATCTCAGCTTGGAGGCTTGAACAGT
Illumina_PE2 SVS.SeqPrimer	GAACCGCTCTTCCGATCTCAGCTTGGAGGCTTGAACA*G*T

*: phosphorothioate bond. iBiodT: internal biotinylated dT. In the barcode assignment primers, HBV-specific sequences are underlined, the SbfI recognition site is double-underlined. The barcode assignment primer used on the internal standard carries a 2-base insertion (red).

Protocol for DNA purification with AMPure XP beads

The following steps were used for DNA purification with AMPure XP beads throughout the protocol:

1. Add the indicated volume of AMPure XP beads to the sample and mix well by pipetting up and down 10 times.

2. Rotate for 5 minutes at room temperature.

3. Perform a quick spin to bring the sample to the bottom of the tube. Place the tube on a magnet for 2 minutes and discard the supernatant.

4. With the tube on the magnet, add 200 µl of freshly prepared 70% ethanol. Wash the beads by rotating the tube 360° on the magnet. Remove the ethanol and repeat the wash one more time.

5. Remove the tube from the magnet and perform a quick spin. Place the tube back on the magnet and remove any remaining ethanol.

6. Dry the tube for 1 min in a 37°C heat block. Do not over-dry the tubes as this will result in bead clumping.

7. Elute the DNA by adding the indicated volume of EB to the beads and mix well by pipetting up and down 10 times. Place the tube on a magnet for 2 minutes and save the supernatant.

8. To minimize sample loss during library preparation, leave the tube on the magnet and transfer the supernatant directly to the reaction in the next step of the protocol (whenever possible). Typically, the maximum volume that can be transferred is 1 µl less than the volume of eluate.

Measure viral load by qPCR

The concentration of "full-length" genomes in each HBV sample is measured by quantitative real-time PCR, using six 10-fold dilutions of a linearized clone of full-length HBV as DNA standards. Quantification is achieved by inference from a standard curve generated using the DNA standards.

1. Generate a 10-fold dilution series of six DNA standards from 10^2 to 10^7 copies/µl using a linearized clone of HBV. Each dilution will be included in triplicate on the qPCR plate.

2. Prepare a master mix of the PCR in a 1.5 mL LoBind tube and mix thoroughly by vortexing. Load each well of the 96-well qPCR plate that will be used for quantitation:

H_2O	8.16 µl
EXPRESS SYBR GreenER qPCR Supermix	10 µl
10 µM HBV_Hong_F	0.4 µl
10 µM HBV_Hong_R	0.4 µl
25 µM ROX	0.04 µl
	19 µl

2. To each well, add 1 µl of the DNA standard or HBV sample (included in triplicate) for a total reaction volume of 20 µl. Seal the qPCR plate and perform a brief centrifugation to collect the reagents in the bottom of the wells.

3. Place the qPCR plate in the real-time PCR machine, programmed with the following protocol:

2:00 at 94ºC	
0:20 at 94ºC	40 cycles
0:30 at 60ºC	
5:00 at 68ºC	
1:00 at 95°C	Melt curve analysis
0:30 at 55°C	
0:30 at 95°C	

6. Generate a standard curve from the DNA standards. Calculate the concentration of the HBV sample by using the average of the triplicate data.

7. Proceed with Step 1 of library preparation.

Important notes before starting:

- Refer to Appendix A for a discussion of quality assessment and troubleshooting of BAsE-Seq libraries.
- We recommend advanced scheduling of the library preparation workflow to plan for stopping points. Suitable stopping points include the end of Steps 2, 4, 5, 6, 7 or 8, and the sample can be stored at -20ºC until the next day. In general, higher yields can be obtained if the number of stopping points is minimized. Our typical workflow includes the following stopping points:

  - Before PCR1 in Step 2
  - Before PCR2 in Step 2
  - End of Step 2
  - Overnight incubation with Dynabeads in Step 5
  - Overnight circularization reaction in Step 7
  - End of Step 7

Step 1: Perform barcode assignment

A unique barcode will be assigned to each strand of the HBV genome using a 2-cycle PCR. A restriction site for SbfI will be introduced at the barcode-proximal end of the genome. The reaction is digested with Exonuclease I and purified with AMPure beads to ensure complete removal of barcoding oligos before Step 2.

1. Set up the following reaction in a 0.2 ml PCR tube. Prepare a master mix, if necessary, and add the HBV DNA at the final step to each tube. Keep the master mix and PCR tubes on ice until the denaturation temperature (94ºC) has been reached on the thermocycler.

10^6 HBV genomes	1.0 µl
H_2O	41.7 µl
10x reaction buffer (15 mM $MgCl_2$)	5.0 µl
10 mM dNTPs	1.0 µl
10 µM A-SbfI-ID20-HBV_For	0.4 µl
10 µM B-HBV-Rev	0.4 µl
Long PCR Enzyme Mix (5U/µl)	0.5 µl
	50 µl

2. Run the following PCR program:

5:00 at 94ºC	
0:45 at 94ºC	2 cycles
4:00 at 60ºC	
7:00 at 68ºC	
4ºC forever	

3. Add 3 µl of Exonuclease I (20U/µl) to the PCR reaction. Vortex thoroughly to mix. Incubate for 1 hour at 37ºC, followed by heat inactivation for 5 minutes at 98ºC. Proceed to the next step immediately.

4. Purify the Step 1 product with 53 µl of AMPure XP beads (bead:DNA ratio = 1). Proceed with AMPure purification and elute the DNA in 50 µl of TE.

Step 2: Clonal amplification of barcode-tagged genomes

Barcode-tagged genomes from Step 1 will be clonally amplified by performing PCR using universal primers. To maximize reaction yield, clonal amplification will involve two stages: PCR1 with high cycle number (>20) to pre-amplify the barcode-tagged genomes, followed by PCR2 with low cycle number (<10) to obtain the final product at high yield. We have observed that over-amplification in PCR1 or PCR2 will result in chimeric PCR products due to inefficient amplification during the later cycles of PCR. To minimize chimerism, the optimal cycle number will be determined separately by performing real-time PCR. See Appendix B for a more detailed discussion.

1. If an internal standard will be included in the library, Step 1 products from the internal standard and clinical sample will be mixed (at the desired ratio) at this step to obtain a combined sample containing 40,000 genomes.

2. Set up the following reactions in a 96-well qPCR plate. Each reaction will be performed in **triplicate** on the qPCR plate. Include a negative control (no template). Seal the qPCR plate and perform a brief centrifugation to collect the reagents in the bottom of the wells.

40,000 genomes	2.0 µl
H_2O	30.9 µl
10x reaction buffer (15 mM $MgCl_2$)	5.0 µl
10 mM dNTPs	1.0 µl
10 µM Uni-A-SbfI-Bio	5.0 µl
10 µM Uni-B	5.0 µl
10x SyBr Green I	0.5 µl
25 µM ROX	0.1 µl
Long PCR Enzyme Mix (5U/µl)	0.5 µl
	50 µl

3. Place the qPCR plate in the real-time PCR machine, programmed with the following protocol:

2:00 at 94ºC	
0:20 at 94ºC	10 cycles
0:30 at 60ºC	10 cycles
4:00 at 68ºC	10 cycles
0:20 at 94ºC	25 cycles
0:30 at 60ºC	25 cycles
4:00 at 68ºC (+2s/cycle)	25 cycles
1:00 at 95°C	Melt curve analysis
0:30 at 55°C	Melt curve analysis
0:30 at 95°C	Melt curve analysis

4. Calculate and plot the average amplification curve for the triplicate reactions in the sample. From the curve, identify the cycle number (x) that is at the halfway point of the log-linear phase of PCR. An example of an amplification plot is shown below. Run a 0.7% agarose gel to verify the presence of a ~3.2 kb PCR product.

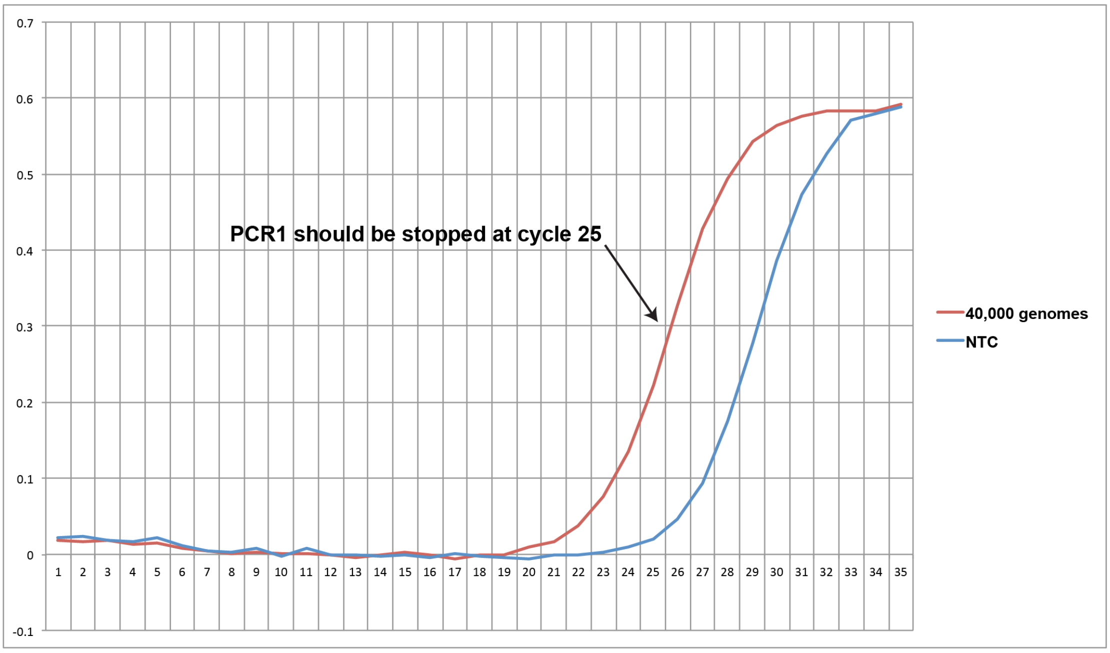

5. After obtaining the optimal cycle number for PCR1, perform a **duplicate** (Rep1 and Rep2) of PCR1 in the standard thermocycler. The product of Rep1 will be used in steps 7-9 for real-time PCR to identify the optimal cycle number for PCR2, and the product of Rep2 will be used in step 10 as template for PCR2.

40,000 genomes	5.0 µl
H_2O	28.5 µl
10x reaction buffer (15 mM $MgCl_2$)	5.0 µl
10 mM dNTPs	1.0 µl
10 µM Uni-A-SbfI-Bio	5.0 µl
10 µM Uni-B	5.0 µl
Long PCR Enzyme Mix (5U/µl)	0.5 µl
	50 µl

2:00 at 94ºC
0:20 at 94ºC } 10 cycles
0:30 at 60ºC
4:00 at 68ºC
0:20 at 94ºC } (x-10) cycles
0:30 at 60ºC
4:00 at 68ºC (+2s/cycle)
4ºC forever

6. Add 35 µl of AMPure XP beads (bead:DNA ratio = 0.7). Proceed with AMPure purification and elute the DNA in 40 µl of EB.

7. Set up the following reactions in a 96-well qPCR plate using Rep1 as a template. Each reaction will be performed in **triplicate** on the qPCR plate. Include a negative control (no template). Seal the qPCR plate and perform a brief centrifugation to collect the reagents in the bottom of the wells.

5,000 pre-amplified genomes from Rep1	5.0 µl
H_2O	27.9 µl
10x reaction buffer (15 mM $MgCl_2$)	5.0 µl
10 mM dNTPs	1.0 µl
10 µM Uni-A-SbfI-Bio	5.0 µl
10 µM Uni-B	5.0 µl
10x SyBr Green I	0.5 µl
25 µM ROX	0.1 µl
Long PCR Enzyme Mix (5U/µl)	0.5 µl
	50 µl

8. Place the qPCR plate in the real-time PCR machine, programmed with the following protocol:

2:00 at 94ºC
0:20 at 94ºC } 10 cycles
0:30 at 60ºC
4:00 at 68ºC
0:20 at 94ºC } 7 cycles
0:30 at 60ºC
4:00 at 68ºC (+2s/cycle)
1:00 at 95°C } Melt curve analysis
0:30 at 55°C
0:30 at 95°C

9. Calculate and plot the average amplification curve for the triplicate reactions in the sample. From the curve, identify the highest cycle number (y) that is still within the log-linear phase of PCR. An example of an amplification plot is shown below.

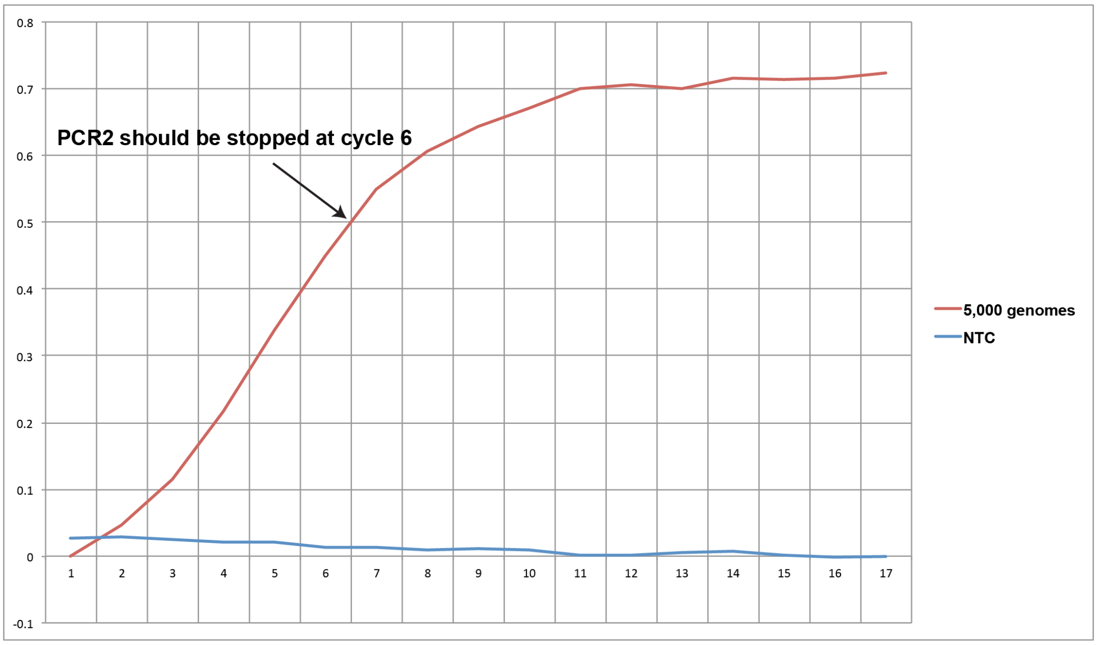

10. Distribute the product of Rep2 across 8 wells of a 0.2 mL PCR strip tube (40,000 genomes in total) and set up the following PCR in the standard thermocycler.

5,000 pre-amplified genomes from Rep2	5.0 µl
H_2O	28.5 µl
10x reaction buffer (15 mM $MgCl_2$)	5.0 µl
10 mM dNTPs	1.0 µl
10 µM Uni-A-SbfI-Bio	5.0 µl
10 µM Uni-B	5.0 µl
Long PCR Enzyme Mix (5U/µl)	0.5 µl
	50 µl

2:00 at 94ºC
0:20 at 94ºC }
0:30 at 60ºC } y cycles
4:00 at 68ºC }
4ºC forever

11. Combine the 8 PCRs into a 1.5 ml LoBind tube and add 280 µl of AMPure XP beads (bead:DNA ratio = 0.7). Proceed with AMPure purification and use 800 µl (instead of 200 µl) of 70% ethanol for the wash steps. Elute the DNA in 45 µl of EB.

12. Measure the concentration of the sample using the dsDNA Qubit BR assay kit.

13. Load 1 µl of the sample on a DNA 7500 chip on the Bioanalyzer to verify the presence of a ~3.2 kb product.

Step 3: Digest with *SbfI*

Clonally amplified genomes from Step 2 will be digested by SbfI to create a 4-bp 3'-overhang at the barcode-proximal end of the amplicon. This will protect the barcode-proximal end of the amplicon from exonuclease activity in Step 4.

Important note: We recommend using 6 µg of Step 2 product for *SbfI* digest at this step to guarantee sufficient yield for the remaining steps of the protocol. However, this may not be possible if the yield for Step 2 is lower than expected. From our experience, we have successfully generated libraries from as little as 2.5 µg of input at this step. The amount of enzyme used in Step 4 will have to be scaled down proportionally.

1. Set up the following reaction in a 0.2 ml tube:

6 µg of Step 2 product	42 µl
10x CutSmart Buffer	5 µl
SbfI-HF (20U/µl)	3 µl
	50 µl

2. Incubate at 37ºC for 1 hour, followed by heat inactivation for 20 minutes at 80ºC. Incubation should be performed in a thermocycler with a heated lid.

3. Transfer the sample to a 1.5 ml LoBind tube and add 90 µl of AMPure XP beads (bead:DNA ratio = 1.8). Proceed with AMPure purification and elute the DNA by adding 53 µl of EB.

Step 4: Unidirectional deletion with Exonuclease III

A broad range of deletions will be generated from the barcode-distal end of each molecule using Exonuclease III, followed by blunt-ending with S1 Nuclease. The barcode-proximal ends of the molecules are protected from exonuclease activity by the 3'-overhang generated in Step 3.

Important note: If the amount of input for Step 3 is less than 6 µg, the amount of Exonuclease III and S1 Nuclease used in this step will have to be scaled down proportionally.

1. For each library, aliquot 3 µl of 0.5M EDTA to each of 5 x 0.2-ml LoBind tubes.

2. Set up the following reaction in a 0.2 ml tube:

Step 3 product	52 µl
Exonuclease III 10x Reaction Buffer	6 µl
	58 µl

3. Pre-warm the tube at 30ºC in a thermocycler for 5 minutes.

4. Using a P2 or P10 pipette, add 2 µl of Exonuclease III, start the timer, then

mix as rapidly as possible using a P100 or P200 pipette set at ‘55 µl’. Take care not to introduce bubbles or splash the reaction on the walls of the tube.

5. At the following 5 time points, remove 12 µl of the reaction and add it to the tubes containing 0.5M EDTA. Mixing thoroughly by pipetting, then perform heat inactivation in a thermocycler at 80ºC for 15 minutes. Leave the Exonuclease III reaction in the 30ºC heat block throughout.

   Time points (min): 1 / 3 / 5 / 7 / 9

6. Combine the 5 samples for each library into a 1.5 mL LoBind tube (75 µl total). Perform purification with 135 µl of AMPure XP beads (bead:DNA ratio = 1.8) and elute the DNA in 42 µl of EB.

7. Dilute the S1 Nuclease to 12 U/µl and mix by vortexing:

S1 Nuclease (89U/µl)	3 µl
10x S1 Nuclease Reaction Buffer	2.225 µl
H_2O	17.025 µl
	22.25 µl

8. Set up the following reaction in a 1.5 mL LoBind tube and mix thoroughly by pipetting:

Exonuclease III digested sample	41 µl
S1 Nuclease 10x Reaction Buffer	5 µl
S1 Nuclease (12 U/µl)	4 µl
	50 µl

9. Incubate at room temperature for 30 minutes. Add 5 µl of 0.5M EDTA to stop the reaction.

10. Add 99 µl of AMPure XP beads (bead:DNA ratio = 1.8). Proceed with AMPure purification and elute the DNA by adding 41 µl of EB.

Step 5: Purify barcode-containing fragments

Biotinylated DNA carrying barcode-tagged fragments will be purified using paramagnetic streptavidin beads.

Capture biotinylated DNA:

1. Resuspend Dynabeads from the Dynabeads kilobaseBINDER kit by shaking or vortexing the vial to obtain a homogeneous suspension.

2. Transfer 10 µl (100 µg) of resuspended beads to a 1.5 mL LoBind tube. Place the tube on a magnet for 2 minutes.

3. Carefully pipette off the supernatant while the tube remains on the magnet. Avoid touching the bead pellet with the pipette tip.

4. Remove the tube from the magnet. Add 40 µl of Binding Solution and gently resuspend by pipetting to avoid foaming.

5. Place the tube on a magnet and remove the supernatant.

6. Resuspend the beads in 40 µl of Binding Solution.

7. Add 40 µl of the Step 4 product to the beads. Mix gently to avoid foaming of the solution.

8. Incubate the tube at room temperature for 15 hours on a rotator to keep the beads in suspension.

9. Place the tube on a magnet for 2 minutes and remove the supernatant.

10. Wash the Dynabeads/DNA-complex twice with 100 µl of Washing Solution.

11. Wash the Dynabeads/DNA-complex once in 1x TE buffer.

Dissociate the DNA:

1. Add 300 µl of QIAGEN PBI buffer to the beads and incubate on a thermomixer at 50°C for 30 minutes at 1400 rpm.

2. Place the tube on a magnet for 2 minutes and transfer the supernatant to a Minelute column.

3. Repeat the procedure by adding another 300 µl of QIAGEN PBI buffer to the beads and incubate on a thermomixer at 50°C for 30 minutes at 1400 rpm.

4. Place the tube on a magnet for 2 minutes and transfer the supernatant to the same Minelute column (600 µl total).

5. Purify the DNA using the Minelute column, following the QIAGEN protocol. Perform the elution step twice, each time with 13.5 µl of EB.

Step 6: End repair

Barcode-tagged fragments from Step 5 will be blunt-ended and 5'-phosphorylated.

1. Set up the following reaction in a 0.2 mL tube:

Step 5 product	25.75 µl
10x T4 DNA ligase buffer (NEB)	4 µl
10 mM dNTPs	0.25 µl
T4 DNA polymerase (3U/µl)	5 µl
T4 Polynucleotide Kinase (10U/µl)	5 µl
	40 µl

2. Incubate at 20ºC for 20 minutes in a thermocycler with a heated lid, followed by heat inactivation at 75ºC for 20 minutes.

3. Add 28 µl of AMPure XP beads (bead:DNA ratio = 0.7). Proceed with AMPure purification and elute the DNA by adding 20 µl of EB.

4. Measure the concentration of the sample using the dsDNA Qubit HS assay kit.

Step 7: Circularize DNA

End repaired DNA from Step 6 will be circularized by intramolecular ligation. After circularization, the distal ends of the genome are brought adjacent to the barcode. To minimize the formation of chimeric circles (formed by a combination of inter- and intra-molecular ligation events), ≤100 ng of template will be used for circularization and the reaction will be carried out at 10°C in a volume of 45 ml. Uncircularized DNA is removed by exonuclease digest. See Appendix C for a more detailed discussion.

1. Prepare the following equipment and reagents:
   - 10°C water bath in a 4°C cold room
   - Nuclease-free H_2O pre-chilled to 4°C
   - 70% ethanol pre-chilled to 4°C

2. Keeping the reagents and reaction tube in a regular ice bath, set up the following reaction in a 50 mL centrifuge tube. Mix the reaction thoroughly by gently inverting the tube 20 times.

≤100 ng of Step 6 product	20 µl
H_2O	40.46 ml
10x T4 DNA Ligase Buffer	4.50 ml
	45 ml

3. Add 25 µl of T4 DNA Ligase and mix the reaction thoroughly by gently inverting the tube 20 times. Place the reaction tube in the 10°C water bath (placed in the cold room) for 16 hours. Alternatively, the reaction can be carried out at 10°C in a cooling block placed at room temperature, such as using the Eppendorf thermomixer R fitted with a 4 x 50 ml thermoblock.

4. Split each reaction between two new 50 mL centrifuge tubes, each containing ~22.5 mL of the reaction (use pouring instead of pipetting to minimize loss). To each tube, add 10 µl of GlycoBlue, 4.5 mL of 3M sodium acetate (pH 5.2) and 22.5 mL of isopropanol. Mix thoroughly by inverting the tubes and store the tubes in -30ºC for at least 1 hour.

5. Centrifuge the tubes at 15,000 x g for 30 minutes at 4ºC.

6. Carefully remove the supernatant with a pipette and wash the pellet with 10 mL of ice-cold 70% ethanol.

7. Centrifuge at 15,000 x g for 10 minutes at 4ºC. Make sure to position the tubes in the same orientation as during the first spin.

8. Carefully remove the supernatant with a pipette and wash the pellet again with 10 mL of ice-cold 70% ethanol.

9. Centrifuge at 15,000 x g for 10 minutes at 4ºC. Make sure to position the tubes in the same orientation as during the previous spin.

10. Carefully remove the supernatant with a pipette. Perform a quick spin (1 minute) and remove the residual ethanol with a fine-tipped pipette.

11. Allow the pellet to air-dry in a laminar flow hood for 15-30 minutes.

12. For each sample, re-suspend the pellets in 35 µl of EB. Use the solution used to resuspend the first pellet to re-suspend the other pellet from the same library such that the final volume of purified sample for each library is ~35 µl.

13. Perform the exonuclease digest in a 0.2 mL tube in a thermocycler to digest any uncircularized DNA. Incubate at 37ºC for 30 minutes followed by 80ºC for 20 minutes.

Purified sample	35 µl
10x Lambda Exo Reaction Buffer	5 µl
Lambda Exonuclease (5U/µl)	8 µl
Exonuclease I (20U/µl)	2 µl
	50 µl

14. Transfer the sample to a 1.5 mL LoBind tube and add 90 µl of AMPure XP beads (bead:DNA ratio = 1.8). Proceed with AMPure purification and elute the DNA by adding 20 µl of EB.

15. Use a SpeedVac to reduce the volume of the sample to 6 µl.

16. Measure the concentration of the sample using the dsDNA Qubit HS assay kit.

Step 8: Random fragmentation and adaptor tagging

Circularized molecules from Step 7 will be fragmented and tagged with Illumina adaptors using the Nextera XT transposome, followed by PCR to add sequences required for cluster generation on Illumina flow cells.

1. Using 1 ng of the Step 7 product, follow the "Tagmentation of Input DNA" step in the Nextera XT Sample Preparation Guide.

2. Set up the following PCR in a 0.2-mL tube using the Nextera PCR Master Mix (NPM) from the Nextera XT kit. However, instead of using the primers provided in the kit, use the custom primers listed below.

Tagmented sample	25 µl
NPM	15 µl
5 µM NexteraXT-PE1	5 µl
5 µM Illumina-PE2_HBV-For.RevComp	5 µl
	50 µl

3. Run the following program in a standard thermocycler (do not follow the program provided in the Nextera XT Sample Preparation Guide):

   3:00 at 72ºC
   0:30 at 95ºC
   0:10 at 95ºC ⎫
   0:30 at 55ºC ⎬ 14 cycles*
   1:00 at 72ºC ⎭
   5:00 at 72ºC
   10ºC forever

   *Additional cycles of PCR will have to be performed if the input for this step is less than 1 ng.

4. Transfer the entire sample to a 1.5 mL LoBind tube and adjust the sample volume to exactly 100 µl by adding ~50 µl of nuclease-free H_2O. Add 65 µl of AMPure XP beads (bead:DNA ratio = 0.65). Proceed with AMPure purification and elute the DNA by adding 25 µl of EB.

5. Load 1 µl of the sample on a DNA High Sensitivity chip on the Bioanalyzer. The expected size distribution of the library is 400 bp to 2000 bp. Calculate the average size of the library by performing a "region analysis" of the 200-1000 bp region. This will be used in Step 9 to calculate the concentration of the library.

Step 9: Library quantification and sequencing

Measure the effective concentration of the library and set up a sequencing run on the MiSeq.

1. Perform real-time PCR using the Library Quantification Kit (KAPA Biosystems) to obtain the concentration of the library.

2. Following manufacturer's instructions, load 18 pM of the library on a MiSeq 300-cycle reagent kit v2. Use a custom sequencing primer "Illumina_PE2 SVS.SeqPrimer" for the second sequencing read.

Appendix A: General troubleshooting and quality assessment

For users that are using the BAsE-Seq protocol for the first time, we recommend using mixed viral clones as input to assess the quality of libraries generated using the protocol. This is because slight changes in reaction conditions or technical variability between different users can affect the baseline error frequency and rate of chimerism (mis-assignment of barcodes to the wrong template sequences) of each library.

A higher-than-expected baseline error frequency can arise for several reasons. First, barcode assignment primers may be incompletely removed after barcode assignment, which can result in extra rounds of barcode assignment in a subsequent PCR step and increase the frequency of PCR errors on barcode-tagged template molecules. To prevent this, we performed Exonuclease I digestion followed by AMPure purification to remove the unused barcode assignment primers at the end of Step 1. During protocol development, we also spiked in a synthetic template that contained a ~1 kb insert flanked by HBV forward- and reverse-priming sequences as an internal negative control during PCR1 in Step 2 to ensure that barcode assignment primers have been completely removed. Second, contamination originating from previous library preparations can result in an inflated error rate due to the accumulation of PCR errors. Thus, it is important to minimize contamination between libraries by maintaining good laboratory practice. We also recommend using a PCR hood with UV sterilization for library preparation. From time to time, it is also a good idea to change the sequences of the barcode assignment primers to prevent carry-over from earlier experiments.

During protocol development, we mixed the two HBV clones at different steps during the BAsE-Seq protocol to identify the steps where molecular chimerism was occurring and assessed their relative contribution to the overall rate of chimerism. For example, two different libraries can be constructed in which the two clones were mixed before Step 1 (barcode assignment) or after Step 2 (clonal amplification of barcode-tagged genomes). If the frequency of chimeric sequences observed in the latter library was significantly lower than the former library, we concluded that molecular chimerism was occurring in Step 1 or 2. Using this approach, we determined that molecular chimerism was most likely to occur in Steps 2 and 7 and performed extensive optimization in both steps to minimize the rate of molecular chimerism (further discussed in Appendix B and C).

For clinical samples, we recommend using an internal standard to estimate the baseline error frequency during library preparation and sequencing. In our protocol, we prepared the internal standard by assigning barcodes separately to HBV Clone-2 (using 'A-SbfI-ID20v2-HBV_For' instead of 'A-SbfI-ID20-HBV_For'); these barcodes contained a 2-base insertion that allowed us to distinguish them from patient-specific viral genomes. After Step 1, the internal standard was mixed with patient-specific viral genomes and used as input for Step 2. Genomes obtained from the internal standard were analyzed for errors and the highest per-base error rate was used to set the baseline error frequency of the library. We used the baseline error frequency as a threshold below which a SNV might be due to an error and above which a SNV was treated as a true SNV.

Appendix B: Step 2 optimization

As described in Appendix A, we had initially observed that a substantial amount of molecular chimerism was occurring in Step 2. We reasoned that this was caused by PCR-induced recombination due to inefficient amplification at later cycles of PCR. More specifically, incomplete extension products from earlier cycles of PCR could be acting as primers on heterologous template molecules, resulting in chimeric PCR products. Thus, we developed a real-time PCR assay to monitor the efficiency of PCR amplification in Step 2. Using the amplification curves (which measure the accumulation of PCR product) from real-time PCR, we observed that PCRs that were stopped during the log-linear phase produced significantly less chimeric sequences compared to PCRs with 40 cycles of amplification when they were used to generate BAsE-Seq libraries (see table below). This observation indicated to us that PCR-induced recombination can be avoided by ensuring that PCR occurred efficiently.

Subsequently, we determined that our protocol required 3 to 6 μg of input for Step 3 and a standard PCR in Step 2 did not provide sufficient yield. Through a series of optimization steps, we determined that a combination of modifications allowed us to increase the yield from Step 2 while staying within the log-linear phase of PCR amplification. First, we performed two PCRs: a pre-amplification (PCR1, with high cycle number) was performed first, followed by a second amplification (PCR2, with low cycle number). The number of cycles performed in PCR1 or PCR2 was decided by performing a separate real-time PCR on a replicate sample. Second, the PCR1 product was cleaned up by AMPure beads and used as template for PCR2. Third, the template for PCR2 was distributed across eight separate PCRs.

**PCR conditions**	**Clone-1 haplotypes**	**Clone-2 haplotypes**	**% Clone-1**[a]
40 cycles	0	2,830	0
Stopped at log-linear phase	448	3,318	11.9

[a]Each library was generated by mixing HBV Clone-1 and Clone-2 at 1:9 ratio before Step 1. The number of haplotypes carrying SNVs that were perfectly concordant for Clone-1 or Clone-2 sequence is shown. If molecular chimerism was occurring during library preparation, our consensus base calling approach for individual genomes will suppress SNV-calls from the less abundant clone (Clone-1). Thus, the proportion of Clone-1 haplotypes serves as a proxy for the level of chimerism in these libraries.

Appendix C: Step 7 optimization

During protocol development, we tried using CircLigase II (Epicentre) to perform circularization of ssDNA templates. Despite extensive efforts to optimize reaction conditions, CircLigase II did not efficiently circularize large templates (>1.2 kb). A similar observation was made in Pan *et al.,* "Two methods for full-length RNA sequencing for low quantities of cells and single cells", *Proc Natl Acad Sci USA* (2013) vol. 110 (2) pp. 594-9. Eventually, we abandoned this approach and focused on using T4 DNA Ligase for circularization of dsDNA templates.

To determine optimal conditions for dsDNA circularization, we developed a qPCR-based assay where two sub-genomic HBV sequences (each ~1 kb long) were mixed at equimolar amounts and used as dsDNA templates for circularization. The qPCR assay included primers that specifically detect both junctions formed by intra-molecular ligation, as well as one primer pair that detects one possible junction formed by inter-molecular ligation. A dilution series of DNA standards was used to estimate the amount of product obtained for each type of junction identified in the assay.

Using this qPCR assay, we screened a large number of reaction conditions and ultimately determined that two key parameters were critical for obtaining a high rate of intra-molecular ligation and a low rate of inter-molecular ligation: reaction volume and temperature. Notably, significant improvements were achieved by increasing the reaction volume to 45 ml or decreasing the reaction temperature to 10°C (as shown below). A combination of both modifications resulted in an intra-molecular ligation rate of ~5%. Subsequently, we constructed BAsE-Seq libraries from mixed HBV clones (using various conditions) and verified that decreasing proportions of chimeric sequences were obtained by increasing reaction volume or decreasing temperature. Ultimately, we determined that by restricting the amount of template for circularization to ≤100 ng, we were able to achieve almost no chimeric sequences in our BAsE-Seq libraries using the protocol.

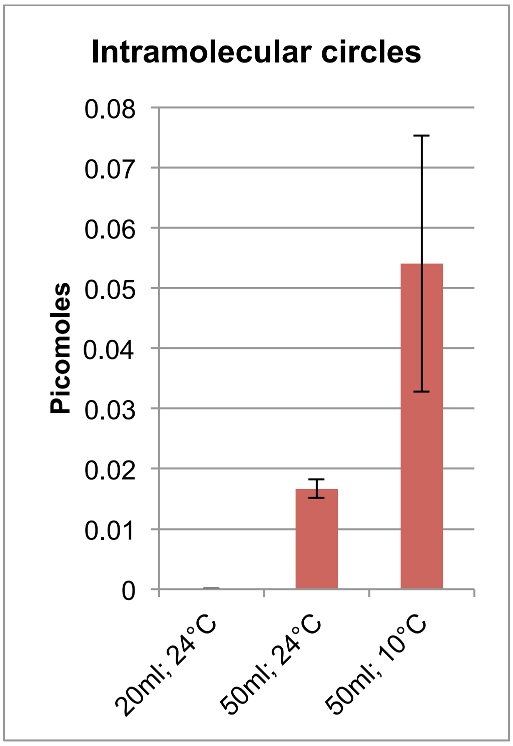

Supplement: Additional file 2: — BAsE-Seq detailed protocol. [file 13059_2014_517_MOESM2_ESM.pdf]
